# Differential MicroRNA Expression Levels in Cutaneous Acute Graft-Versus-Host Disease

**DOI:** 10.3389/fimmu.2018.01485

**Published:** 2018-07-10

**Authors:** Sadaf Atarod, Jean Norden, Louis A. Bibby, Anne Janin, Philippe Ratajczak, Clare Lendrem, Kim F. Pearce, Xiao-Nong Wang, Steven O’Reilly, Jacob M. Van Laar, Matthew Collin, Anne M. Dickinson, Rachel E. Crossland

**Affiliations:** ^1^Haematological Sciences, Institute of Cellular Medicine, Newcastle University, Newcastle upon Tyne, United Kingdom; ^2^Newborn Medicine, Brigham and Women’s Hospital, Harvard University, Boston, MA, United States; ^3^Université Paris Diderot, INSERM, UMR_S1165, Paris, France; ^4^Faculty of Health and Life Sciences, Northumbria University, Newcastle upon Tyne, United Kingdom; ^5^Department of Rheumatology and Clinical Immunology, University Medical Centre Utrecht, Utrecht University, Utrecht, Netherlands

**Keywords:** microRNA, GvHD, biomarker, molecular profiling, cutaneous

## Abstract

Allogeneic hematopoietic stem cell transplantation is a curative treatment for numerous hematological malignancies. However, acute graft-versus-host disease (aGvHD) is a major complication affecting 40–70% of all transplant patients, whereby the earliest and most frequent presentation is in the skin. MicroRNAs play a role in varied biological process and have been reported as potential biomarkers for aGvHD. More recently, microRNAs have received added attention as circulatory biomarkers that can be detected in biofluids. In this study, we performed global microRNA expression profiling using a discovery cohort of diagnostic cutaneous aGvHD biopsies (*n* = 5, stages 1–3) and healthy volunteers (*n* = 4), in order to identify a signature list of microRNAs that could be used as diagnostic biomarkers for cutaneous aGvHD. Candidate microRNAs (*n* = 8) were then further investigated in a validation cohort of post-HSCT skin biopsies (*n* = 17), pre-HSCT skin biopsies (*n* = 6) and normal controls (*n* = 6) for their association with aGvHD. Expression of let-7c (*p* = 0.014), miR-503-5p (*p* = 0.003), miR-365a-3p (*p* = 0.02), miR-34a-5p (*p* < 0.001) and miR-34a-3p (*p* = 0.006) were significantly differentially expressed between groups and significantly associated with survival outcome in post-HSCT patients (miR-503-5p ROC AUC = 0.83 *p* = 0.021, Log Rank *p* = 0.003; miR-34a-3p ROC AUC = 0.93, *p* = 0.003, Log Rank *p* = 0.004). There was no association with relapse. A statistical interaction between miR-34a-3p and miR-503-5p (*p* = 0.016) was diagnostic for aGvHD. Expression levels of the miR-34a-5p protein target p53 were assessed in the epidermis of the skin, and an inverse correlation was identified (*r*^2^ = 0.44, *p* = 0.039). Expression of the validated candidate microRNAs was also assessed at day 28 post-HSCT in the sera of transplant recipients, in order to investigate their potential as circulatory microRNA biomarkers. Expression of miR-503-5p (*p* = 0.001), miR-34a-5p (*p* = 0.005), and miR-34a-3p (*p* = 0.004) was significantly elevated in the sera of patients who developed aGvHD versus no-aGvHD (*n* = 30) and miR-503-5p was associated with overall survival (OS) (ROC AUC = 0.80, *p* = 0.04, Log Rank *p* = 0.041). In conclusion, this investigation reports that microRNA expression levels in clinical skin biopsies, obtained at the time of cutaneous aGvHD onset, show potential as diagnostic biomarkers for aGvHD and as predictive biomarkers for OS. In addition, the same microRNAs can be detected in the circulation and show predictive association with post-HSCT outcomes.

## Introduction

Skin is the largest organ of the human body, and it is more frequently affected by acute graft-versus-host disease (aGvHD) (1). Cutaneous aGvHD manifests as a sequence of events that can be divided into three phases. Phase I of cutaneous aGvHD involves the activation of resting Langerhans cells, due to the release of cytokines from damaged host tissues during the chemotherapy regimen and/or irradiation therapy. Phase II of cutaneous aGvHD occurs post allogeneic hematopoietic stem cell transplantation (allo-HSCT) when donor T-cells recognize the antigens presented by activated host dendritic cells (DCs). During phase III, immature keratinocytes undergo apoptosis due to the “cytokine storm,” which exacerbates histological skin damage (2). Cutaneous aGvHD severity is determined by trained independent histopathologists (3).

Currently, biomarkers that can be reliably used in the clinic to stratify patients for improved treatment response, diagnose aGvHD, or predict patient survival outcome are absent. Recently, the roles of several microRNAs have been investigated in aGvHD. miR-146a has been shown to be downregulated in severe-aGvHD murine models, and its target Traf6 elevated, leading to activation of the NFκB pathway (4). Likewise, elevated levels of miR-155 were expressed in the gut of severe-aGvHD patients when compared with normal biopsies (5). miR-34a has been shown to be expressed at higher levels post-transplant in the gut of patients with aGvHD grades II–IV compared with patients with grades 0–I (6). miR-100 expression was shown to be increased in the gut of mice without aGvHD, therefore suggesting a protective function (7). A predictive signature list of microRNAs (miR-423, miR-199a-3p, miR-93*, and miR-377) for aGvHD has previously been detected and validated in the plasma/sera of allo-HSCT patients (8, 9). These microRNAs were able to discriminate patients who had developed aGvHD from those who had not (non-aGvHD group). The microRNAs were associated with reduced overall survival (OS) and aGvHD severity (8). Circulatory microRNAs have also been globally profiled in the context of aGvHD (10) and tissue-specific microRNA expression patterns have been examined in a rat model of aGvHD (11). Circulatory microRNAs show great promise as potential biomarkers, due to the ease and non-invasive nature of sample collection.

In this study, in the discovery cohort, skin biopsies were collected from normal controls, as well as pre-transplant and post-transplant patients at the time of aGvHD disease onset. The samples were analyzed to determine the diagnostic potential of microRNAs in cutaneous aGvHD and their prognostic potential for predicting patient survival. Global microRNA profiling using RT-qPCR was performed to identify a signature microRNA list, which was then further validated in a larger patient validation cohort. Expression of miR-503-5p, miR-34a-5p, miR-34a-3p, and let-7c-5p was associated with aGvHD incidence and survival. Results showed that miR-34a-5p was the most promising microRNA for determining aGvHD severity, based on its significant differential expression levels across all the comparative groups. The protein targets (p53 and c-Myc) of miR-34a-5p were investigated by immunohistochemistry and showed association with expression of miR-34a-5p and skin aGvHD stage. Studies have shown the existence of a positive feedback loop between p53 and miR-34a (12). Likewise, c-Myc expression is suppressed by miR-34a (13). Interestingly, c-Myc has numerous functions in hematopoiesis such as regulating hematopoietic stem cell regeneration and differentiation (14). c-Myc protein is a transcription factor that is constitutively expressed in many tumors and impacts microRNA biogenesis steps in the nucleus (15). Moreover, c-Myc, p53, and miR-34a are all involved in the p53 pathway and the epithelial–mesenchymal transition network (16). In the context of allo-HSCT, p53-deficient mice that develop aGvHD have a better OS when compared with mice with at least one copy of the gene (17). Finally, analysis of the expression of candidate microRNAs was assessed in non-invasive serum samples, in order to evaluate their circulatory biomarker capacity. This would be of great potential clinical benefit, as biofluid samples are preferable to using invasive diagnostic skin biopsies.

Overall, the aims of this investigation were (1) to identify a list of microRNAs with significant potential to diagnose cutaneous aGvHD and to explore their impact on their predicted protein targets, (2) to predict OS and risk of relapse, (3) to further explore the circulatory biomarker potential of candidate microRNAs.

## Materials and Methods

### Patient Criteria

Research was granted ethical approval by the Newcastle and North Tyneside Research Ethics Committee (REC Ref: 14/NE/1136 and 07/H0906/131). Participants gave full informed written consent for their samples to be used for research purposes. Skin biopsies were obtained from allo-HSCT patients pre- (*n* = 6) and post-transplantation (*n* = 17) as well as from healthy volunteers (*n* = 6) with informed written consent. Skin histopathological aGvHD stage was evaluated by two independent histopathologists who were blinded to the origin of the sample, using the Glucksburg (3) criteria. All skin biopsies were collected pre-transplantation and at the time of aGvHD onset. Blood samples were collected from HSCT patients (*n* = 30) at day 28 (D28) post-HSCT in 7-ml vacutainers containing no anti-coagulant from patients undergoing allo-HSCT (years 2009–2013). Clinical details of the cohorts are shown in Tables 1–3. To verify that differential microRNA expression levels were only due to either aGvHD or a result of transplantation, stringent criteria were set that excluded any patient who had received donor-lymphocyte infusion, had cyclosporine withdrawal, had received steroids, or had developed late onset aGvHD (post-100 days from the date of transplantation). Thus, only patients with classic aGvHD (allo-HSCT to onset ≤100 days) were included in all analyses.

**Table 1:** Patient characteristics for the discovery cohort global microRNA skin profiling analysis.

Patient characteristics	A1292	A2224	A2274	A1300	A2137
Diagnosis	NHL	MDM	ALL	CLL	AML
Age	63	58	19	61	66
Transplant type	MUD	MUD	SIB	MUD	MUD
Source	PBSC	PBSC	PBSC	PBSC	PBSC
Protocol	Flu Mel	Flu Mel	TBI Cy	Flu Mel	Flu Mel
Campath protocol (mg)	60	90	30	60	60
RIC	Yes	Yes	No	Yes	Yes
Status at Tx	CR1	CR1	CR1	CR	CR2
CMV patient	−	+	+	+	−
CMV donor	−	−	+	+	+
Patient sex	M	M	M	M	M
Donor sex	M	F	F	M	M
Skin histopathological stage	2	3	2	1	1
Days from Tx (aGVHD onset)	23	18	79	76	33
Overall clinical aGVHD grade	II	IV	II	I	II
Chronic GVHD	N/A	Yes	Yes	Yes	Yes
Relapse	No	No	No	No	No

*Skin biopsies (*n* = 5) were taken from allo-HSCT patients. Exiqon miRCURY LNATM Universal Reverse Transcription microRNA Human Panel I and II were used for the global profiling analysis*.

*AML, acute myeloid leukemia; MDS, myelodysplastic syndrome; CLL, chronic lymphocytic leukemia; NHL, non-Hodgkin’s lymphoma; ALL, acute lymphocytic leukemia; SIB, sibling transplant; MUD, matched unrelated donor; PBSC, peripheral blood stem cells; Flu, fludarabine; Mel, melphalan; Alem, alemtuzumab; Tx, transplant; RIC, reduced intensity conditioning; CR1, complete remission 1; CR2, complete remission 2; CR, complete remission; CMV, cytomegalovirus; M, male; F, female; +, positive; −, negative, chronic GVHD N/A, not applicable as patient died before assessment; allo-HSCT, allogeneic hematopoietic stem cell transplantation*.

**Table 2:** Patient characteristics for the skin validation cohort.

Cohort characteristics		Skin validation cohort Histopathological stage (*n* = 17)				
		0–1 (*n* = 10)		2–3 (*n* = 7)		Difference (*p*-value)
		No.	%	No.	%	
Patient age (median years)		47 (19–65)				

Patient sex	Female	2	67	1	33	1.000
	Male	8	57	6	43	

Donor sex	Female	3	60	2	40	1.000
	Male	7	58	5	42	

Graft source	BM	1	100	0	0	1.000
	PBSC	9	56	7	44	

Underlying disease	ALL	1	50	1	50	0.907
	AML	1	33	2	67	
	MDS	3	75	1	25	
	MF	0	0	1	100	
	NHL	3	60	2	40	
	CLL	1	100	0	0	
	MM	1	100	0	0	

Regimen	Myeloablative	1	33	2	67	0.537
	RIC	9	64	5	36	

Protocol	Cy TBI Alem	1	33	2	67	0.481
	Flu Bus Alem	1	33	2	67	
	Flu Mel Alem	8	73	3	27	

Campath	30 mg	3	60	2	40	1.000
	60 mg	6	60	4	40	
	90 mg	1	50	1	50	

Relationship	SIB	2	50	2	50	1.000
	MUD	8	62	5	38	

Patient CMV status	Negative	4	57	3	43	1.000
	Positive	6	60	4	40	

Donor CMV status	Negative	6	60	4	40	1.000
	Positive	4	57	3	43	

HLA class I compatibility	None	8	67	4	33	0.593
	One	2	40	3	60	

HLA class II compatibility	None	3	50	3	50	0.219
	One	3	43	4	57	
	Two	4	100	0	0	

Survival status	Alive	7	78	2	22	0.153
	Deceased	3	38	5	62	

Relapse status	No	6	55	5	45	1.000
	Yes	4	67	2	33	

Disease status at transplant	CR	1	100	0	0	0.586
	CR1	4	50	4	50	
	CR2	4	80	1	20	
	PR	1	33	2	67	

*Histopathological aGvHD stage was used for patient groupings. Fisher’s exact test was used to estimate the difference in frequencies between the aGvHD groups*.

*BM, bone marrow; PBSC, peripheral blood stem cells; ALL, acute lymphocytic leukemia; AML, acute myeloid leukemia; MDS, myelodysplastic syndrome; MF, myelofibrosis; NHL, non-Hodgkin’s lymphoma; CLL, chronic lymphocytic leukemia; MM, multiple myeloma; RIC, reduced intensity conditioning; Cy, cytarabine; TBI, total body irradiation; Alem, alemtuzumab; Flu, Fludarabine; Bus, Busulfan; Mel, Melphalan; SIB, Sibling; MUD, Matched unrelated donor; CMV, Cytomegalovirus; CR (1-2), Complete remission; PR, Progression*.

**Table 3:** Patient characteristics for the serum cohort.

Cohort characteristics		Serum cohort Overall aGvHD grade (*n* = 30)				
		0 (*n* = 13)		I–III (*n* = 17)		Difference (*p*-value)
		No.	%	No.	%	
Patient age (median years)		50 (23–66)				

Patient sex	Female	3	33	6	67	0.691
	Male	10	48	11	52	

Donor sex	Female	4	44	5	56	1.000
	Male	9	43	12	57	

Graft source	BM	0	0	1	100	1.000
	PBSC	13	45	16	55	

Underlying disease	ALL	1	50	1	50	0.985
	AML	3	37	5	63	
	MDS	3	43	4	57	
	HD	1	50	1	50	
	NHL	3	33	6	67	
	CLL	2	100	0	0	

Regimen	Myeloablative	0	0	3	100	0.238
	RIC	13	48	14	52	

Protocol	Cy TBI Alem	0	0	1	100	0.677
	Flu Bus Alem	2	50	2	50	
	Flu Mel Alem	10	50	10	50	
	Other	1	25	4	75	

Campath	30 mg	NK	NK	NK	NK	NK
	60 mg	NK	NK	NK	NK	
	90 mg	NK	NK	NK	NK	

Relationship	SIB	3	43	4	57	1.000
	MUD	10	43	13	57	

Patient CMV status	Negative	8	44	10	56	1.000
	Positive	5	63	7	27	

Donor CMV status	Negative	10	48	11	52	0.691
	Positive	3	33	6	67	

HLA class I compatibility	None	NK	NK	NK	NK	NK
	One	NK	NK	NK	NK	

HLA class II compatibility	None	NK	NK	NK	NK	NK
	One	NK	NK	NK	NK	
	Two	NK	NK	NK	NK	

Survival status	Alive	6	38	10	62	0.713
	Deceased	7	50	7	50	

Relapse status	No	4	25	12	75	0.064
	Yes	9	50	5	50	

Disease status at transplant	CR	2	40	3	60	0.847
	CR1	5	56	4	44	
	CR2	4	36	7	64	
	PR	2	40	3	60	

*Overall clinical aGvHD grade was used for patient groupings. Fisher’s exact test was used to estimate the difference in frequencies between the aGvHD groups*.

*BM, Bone marrow; PBSC, Peripheral blood stem cells; ALL, Acute lymphocytic leukemia; AML, Acute myeloid leukemia; MDS, Myelodysplastic syndrome; HD, Hodgkins disease; NHL, Non-Hodgkin’s lymphoma; CLL, Chronic lymphocytic leukemia; RIC, Reduced intensity conditioning; Cy, Cytarabine; TBI, Total body irradiation; Alem, Alemtuzumab; Flu, Fludarabine; Bus, Busulfan; Mel, Melphalan; SIB, Sibling; MUD, Matched unrelated donor; CMV, Cytomegalovirus, CR (1-2), Complete remission; PR, Progression; NK, not known*.

### Total RNA Extraction

Skin biopsies (1–4 mm) collected from patients and healthy volunteers were stored in RNA later buffer at −20°C until RNA extraction. Total RNA was extracted as per the manufacturer’s protocol from clinical skin biopsies using the *mir*Vana microRNA Isolation kit (Life Technologies, USA). Blood samples were left to clot, the supernatant centrifuged at 500 × *g* for 5 min and stored at −80°C. Serum aliquots were centrifuged at 4,500 × *g* for 15 min to remove platelets before use. Total RNA was extracted using the NORGEN BioTek^®^ Total RNA Purification Kit, from 250 µl of serum. Extraction was performed in accordance with supplier’s guidelines, with lysis Buffer RL mixed with β-mercaptoethanol 100:1 throughout. The RNA was eluted in 50 µl of elution-solution A and immediately stored at −80°C.

### Quantitative RT-PCR

Global microRNA profiling in the skin samples was performed by The Exiqon MicroRNA qPCR Service. Following three initial quality control checks to assess RNA integrity, purity, and detect PCR inhibitors, 40 ng of total RNA was reverse transcribed to cDNA as per the miRCURY LNA™ Universal RT microRNA PCR protocol. Each cDNA was diluted 100× and then run on both Human panel I (372 microRNAs) and Human panel II (367 microRNAs), equating to *n* = 739 microRNAs, *n* = 3 inter-plate calibrators, *n* = 6 reference controls and one control set (RNA spike-in). None of the samples were pre-amplified. This was followed by the qPCR step which was performed on a Roche LightCycler 480. A total of 186/739 microRNAs (25%) were detectable in all the samples in this cohort. For statistical calculations, 245 microRNAs with expression levels of *C*_q_ < 35 detected in at least three skin biopsies per group were included. The mean of the 186 detected microRNAs (with *C*_q_ < 31) was used to normalize individual assay expression. For the validation study, universal cDNAs were synthesized using locked nucleic acid primers and universal cDNA master mix (Exiqon, Denmark; Product No: 203301) as per the manufacturer’s protocol. Thermal cycler (Applied Biosystems, 2720 Thermal Cycler) conditions comprised of 42°C for 60 min, 95°C for 5 min, and then samples were stored at −4°C until use. The RT-PCR reaction step consisted of microRNA-specific forward and reverse primers (see Table S1 in Supplementary Material) to amplify individual microRNA of interest. ExiLENT SYBR^®^ Green (Product No: 203402) was used for the detection of amplified microRNA products, which was followed by melting curve analysis upon standard qPCR completion as per the manufacturer’s protocol. Normfinder was used to determine the most suitable microRNA for normalization of the validation data (18). miR-103-3p and SNORD48 were used as endogenous controls, with analysis based on the geometric mean for normalization of the expression results.

For serum samples, microRNA-specific cDNA was generated using the TaqMan^®^ MicroRNA Reverse Transcription Kit (Applied Biosystems^®^) and TaqMan^®^ MicroRNA Assays (Applied Biosystems^®^), according to the supplier’s instructions. Each reverse transcription contained 5 µl of total RNA and heating was performed according to supplier recommended cycling conditions. MicroRNA-specific qRT-PCR reactions comprised of TaqMan^®^ MicroRNA Assays (Applied Biosystems^®^) and SensiFAST™ Probe Hi-Rox Reagent (Bioline). Each 10 µl reaction was carried out in triplicate. MicroRNA expression was normalized to two previously identified endogenous controls (U6 and HY3) (19). Reactions were cycled using the 7900 Real-Time PCR System (Applied Biosystems^®^), using manufacturer’s recommended cycling conditions.

### Detection of c-Myc and p53

Pre- (*n* = 5) and post- (*n* = 17) allo-HSCT skin biopsies were sectioned and stained for c-Myc (Abcam, Epitomics at 1:10 dilution) and p53 (Ventana, commercially supplied dilution) proteins using standard formalin-fixed paraffin-embedded immunohistochemistry method (Histopathology Laboratory, Royal Victoria Infirmary, Newcastle upon Tyne).

### Quickscore Method for Assessment of Immunohistopathological Samples

The “quick score method” is a semi-quantitative method (20), used for assessing the percentage of positively stained cells for both p53 and c-Myc proteins. A consensus score was calculated as each section was assessed by two independent researchers, who were blinded to the origin of the samples. The score for each biopsy was calculated by considering both the intensity and the proportion of cells (see Table S2 in Supplementary Material) within that intensity [Quickscore formula = (Intensity 0 × Proportion) + (Intensity 1 × Proportion) + (Intensity 2 × Proportion) + (Intensity 3 × Proportion)]. The whole section was used for calculating the percentage positivity scores. Since miR-34a expression was quantified in both the epidermis and dermis, quick scores were calculated (1) for the entire section regardless of the skin layer and (2) in each skin layer individually.

### Statistical Analysis

Unsupervised hierarchical clustering analysis was performed using RStudio™ Version 0.98.501 (RStudio, Inc., USA). The global profiling results were analyzed using one-way ANOVA on SPSS version 21 (IBM, SPSS Inc., USA). Quantitative RT-PCR results were processed using SDS 2.4 (Life Technologies, USA, v2.4). Differential expression of microRNAs in skin biopsies between all groups (controls, pre-transplant, grades 0–I, and grades II–III/IV) was determined using Kruskal–Wallis one-way analysis of variance with Dunn’s *post hoc* test on GraphPad Prism 5 (GraphPad Software, Inc., USA). Significance was set at *p* < 0.05. Differential expression of microRNAs in serum samples between two groups was assessed using Mann–Whitney *U* test on GraphPad Prism 5. To determine trends in the expression levels for aGvHD severity, the Jonckheere–Terpstra test (SPSS version 21) was also performed for the microRNAs in the signature list. Generalized linear models (ordinal logistic regression) (GLMs) were performed to find the significance and statistical interactions between microRNAs in the signature list using SPSS version 21 (IBM, SPSS Inc., USA). The main effect is statistically defined as an effect of an independent variable on the outcome, while an interaction is a measure of two or more independent variables on the dependent outcome (21). In this scenario, the main effect was the effect of the individual miRNA expressions or significant clinical risk factors on aGvHD outcome and the interaction was a measure of the effect of one independent miRNA expression level as it differed at every condition in comparison to the other independent miRNA expression levels (21). In order to predict aGvHD outcome from a list of covariates, GLMs were built using the binary and/or ordinal logistic regressions. The equation for the binary or ordinal regression models that was used for determining aGvHD grade (outcome) was aGvHD grade (cumulative logit function) = β0 + β1 (miRNA expression 1) + β2 (miRNA expression 2) + β3 (miRNA expression 1 × miRNA expression 2). β0 = intercept coefficient and β1–3 = the coefficient of each miRNA expression. Rotational 3D scatterplots were generated using JMP^®^ Version 11 (SAS Institute Inc., Cary, NC, USA, 1989–2007). Variable screening for both clinical risk factors (patient age, donor–patient relationship, patient and donor gender, CMV status, graft source, underlying disease, mismatches in HLA Class I, and skin histopathological aGvHD grade) and microRNAs was performed using univariate Cox regression analyses. Variables considered as associated with outcome (*p* < 0.2) were entered as candidates in a stepwise multivariate analysis. Receiver operating characteristic (ROC) analysis was performed using survival status as the binary state (classification) variable and microRNA expression on a continuous scale as the test variable, to determine area under the curve (AUC) (SigmaPlot v12.5). OS was calculated using the time in months from transplant to death or last follow-up (October 2014). Survival plots were generated using the Kaplan–Meier (K.M) method and differences in outcome were assessed for significance using the Log-Rank (L.R) test (SPSS v21). The threshold to determine dichotomies for microRNA expression (low and high expression) was evaluated by ROC analysis.

## Results

### Discovery Cohort: Distinct MicroRNA Expression Clusters Differentiate Between Post Allo-HSCT and Control Skin Biopsies

The characteristics of the discovery transplant patient cohort (*n* = 5) for which skin biopsies were profiled are provided in Table 1. The group comprised of male patients with heterogeneous underlying diseases and median age 61 years (range: 19–66) who had undergone allo-HSCT between 2010 and 2012. All the patients received peripheral blood stem cells (PBSC) as the graft. Four patients had reduced conditioning regimens (Fludarabine and Melphalan), while one had myeloablative treatment (total body irradiation and cyclophosphamide). All five patients were in complete remission at the time of transplantation. Overall clinical aGvHD (grades I–IV) developed with a median onset of 33 days (range: 18–79). All patients had matched unrelated donor transplants, except for the youngest patient (19 years old), who had a sibling donor and myeloablative conditioning. Four patients developed chronic GvHD (cGvHD) >100 days after transplantation, and one patient had died before cGvHD assessment. None of the patients relapsed. This heterogeneity was representative of the allo-HSCT clinic. Four healthy volunteers were included in this discovery cohort.

**Figure 1:**
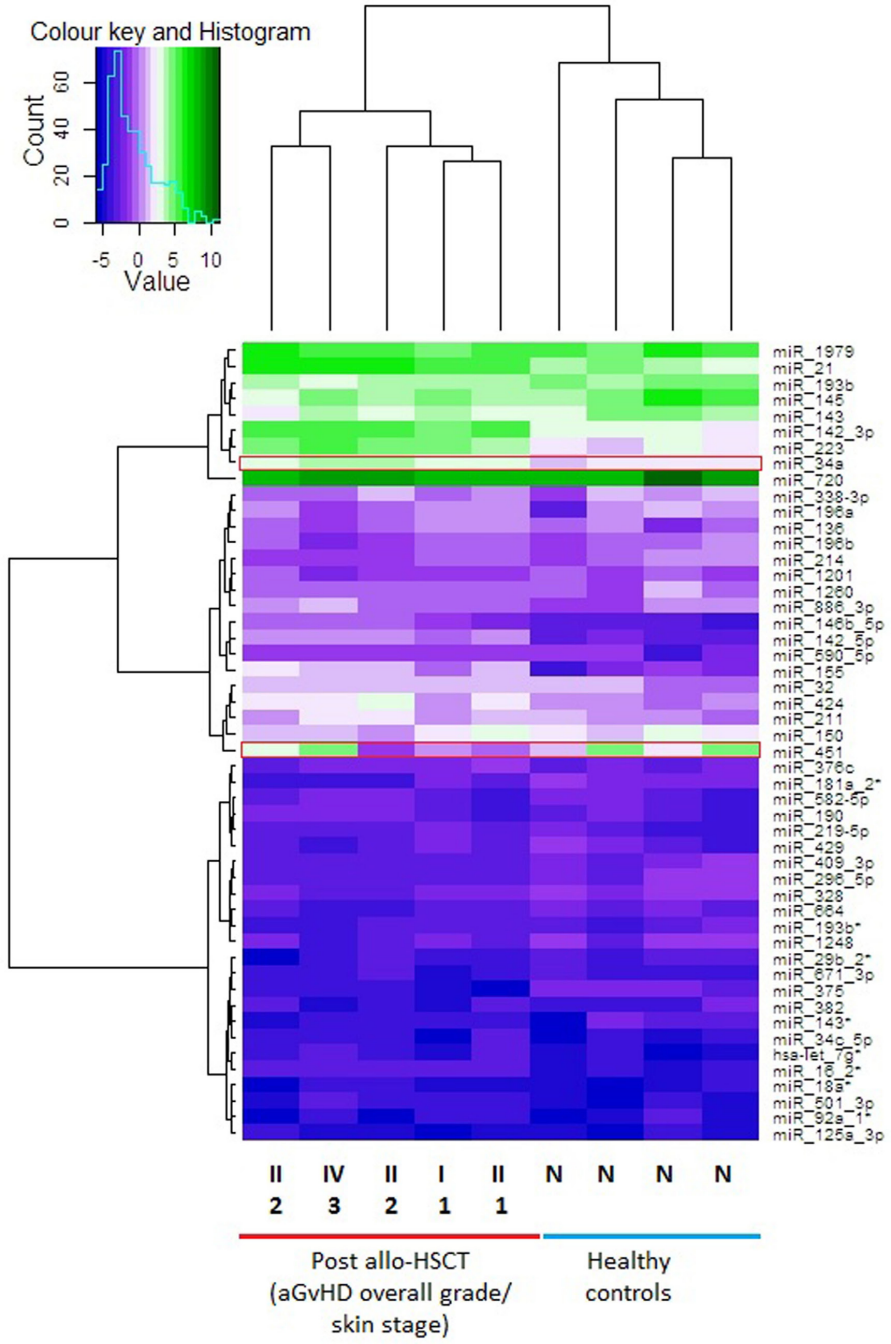
Heat map showing skin biopsy discovery cohort global microRNA expression levels. Each row is representative of one microRNA and each column represents one sample. Unsupervised hierarchical clustering analysis was performed using the expression levels from *n* = 186 detectable microRNAs in the total discovery cohort (*n* = 9). The heatmap shows the top 50 microRNAs that were expressed in all 9 samples. The vertical dendrogram indicates three distinct microRNA clusters, based on the expression values in all samples. The scale of the dendrogram shows the distance, representing similarity between clusters. The horizontal dendrogram shows the clustering of samples into two clusters. Normalized log_2_ values were used for this analysis. The average linkage method was used for the unsupervised hierarchical clustering of the normalized expression results. Fold change range is from −5 to 10. Red boxes highlight microRNAs of interest. Over expressed = green cells, intermediate = light purple cells, under-expressed = dark purple cells. Post allo-HSCT samples are also labeled with their histopathological skin aGvHD stage (1–3) and overall clinical aGvHD grade (II–IV). Abbreviations: N, normal healthy control; allo-HSCT, allogeneic hematopoietic stem cell transplantation; aGVHD, acute graft-versus-host disease.

In the discovery cohort, skin samples with similar microRNA expression values were grouped by performing unsupervised hierarchical clustering. Only microRNAs that were expressed in all skin biopsies, including the healthy control biopsies (*n* = 186), were included in the final analysis. MicroRNA expression in the skin biopsies was used to create a dendrogram and heatmap (Figure 1). The microRNA expression profiles showed considerable patient-to-patient variation, as depicted by the height of the branches, but the most pronounced partition on the dendrogram led to segregation of the skin biopsies into two distinct clusters: the healthy control group in one branch and the allo-HSCT group in the other (Figure 1). The heatmap showed three distinct expression regions, whereby highly upregulated microRNAs clustered in the top branch, and the downregulated microRNAs clustered in the lower section of the heat map. miR-720 was highly expressed in all nine skin biopsies (Mean ΔCp = 9.10) (Figure 1) and has been reported to be an important regulator of T cell proliferation (22). miR-34a-5p expression was significantly different in the healthy controls compared with the allo-HSCT group [control mean ΔCp = 1.97 (range = 1.74–2.25), allo-HSCT mean ΔCp = 3.30 (range = 2.57–4.07); *p* < 0.001] (Figure 1). miR-451-5p is highly expressed in erythrocytes (23) and its levels were highly variable in all the skin biopsies (range = −1.33 to 4.90, SD = 2.28) (Figure 1). This could be attributed to the differences in the vascular network present in each skin biopsy.

A supervised analysis was performed using the discovery cohort of skin biopsies to compare microRNA expression levels between healthy controls, skin aGvHD stages 0–1 (*n* = 2) and skin aGvHD stages 2–3 (*n* = 3). Eight microRNAs (miR-503-5p *p* < 0.001, miR-21-3p *p* < 0.001, miR-34a-5p *p* < 0.001, let-7c-5p *p* < 0.001, miR-142-3p *p* = 0.005, miR-365a-3p *p* < 0.001, miR-23b-3p *p* = 0.001, and miR-24-3p *p* = 0.017) were selected for further validation by RT-qPCR, based on their differential expression levels between control and skin aGvHD groups (Figure S1 in Supplementary Material).

### Validation Cohort: let-7c-5p, miR-503-5p, miR-365a-3p, miR-34a-5p, and miR-34a-3p Are Differentially Expressed in Post Allo-HSCT Skin Biopsies

For the validation study, skin biopsies were taken from transplant patients pre-HSCT (*n* = 6) and post-HSCT (*n* = 17). Characteristics of the post-HSCT patients are shown in Table 2. The cohort comprised of 3 females and 14 males, with biopsy confirmed histopathological skin aGvHD stage 0 *n* = 3, stage 1 *n* = 7, stage 2 *n* = 6, stage 3 *n* = 1, and stage 4 *n* = 0. The median age was 47 years (range: 19–65). Only one patient had a bone marrow infusion, while the remainder (*n* = 16) received PBSC. Three patients had undergone total body irradiation. Median overall clinical aGvHD onset (grade 0 *n* = 1, grade I = 5, grade II = 8, grade III *n* = 1, grade IV *n* = 1) was 23 days (range: 13–98) post allo-HSCT. In all, *n* = 5 (29.4%) of the patients had relapsed and *n* = 8 (47.1%) had died post allo-HSCT at the time of last follow-up. Pre-HSCT skin biopsies (*n* = 6) were taken from patients 7 days prior to transplantation and before the initiation of the conditioning regimen. The patients consisted of 3 females and 3 males of median age 46 years (range: 28–65) of heterogeneous underlying disease [Hodgkin’s disease (*n* = 1), chronic lymphocytic leukemia (*n* = 1), non-Hodgkin’s lymphoma (*n* = 1), acute myeloid leukemia (*n* = 1), and myelodysplastic syndrome (*n* = 2)].

**Figure 2:**
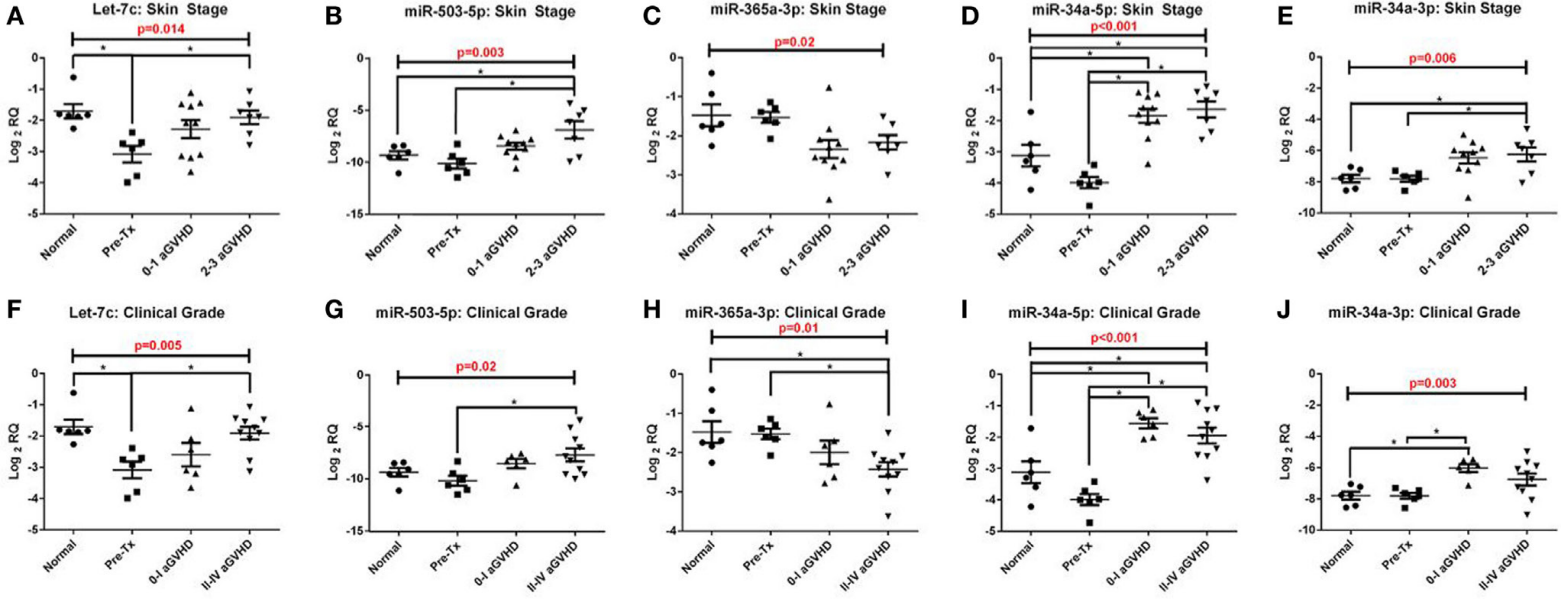
Let-7c-5p, miR-503-5p, miR-365a-3p, miR-34a-5p and miR-34a-3p expression in the validation cohort skin biopsies. MicroRNA expression results for **(A,F)** let-7c-5p, **(B,G)** miR-503-5p, **(C,H)** miR-365a-3p, **(D,I)** miR-34a-5p and **(E,J)** miR-34a-3p were analysed on the basis of **(A–E)** histopathological skin aGvHD stage (0–3, *n* = 17) and **(F–J)** the overall aGvHD clinical grade (0–IV, *n* = 16). Kruskal-Wallis analysis of variance was used to determine the significance, set at *p* ≤ 0.05 (*). Scatterplots show the mean and SEM. Abbreviations: Normal, healthy controls; Pre-Tx, Pre-transplant.

In the validation cohort, RT-qPCR results confirmed that five of the eight target microRNAs identified in the discovery cohort were significantly differentially expressed across the four groups analyzed [pre-transplant (*n* = 6), healthy control (*n* = 6), grades 0–I aGvHD (*n* = 10), and grades II–III aGvHD (*n* = 7)], according to histopathological skin aGvHD stage [let-7c-5p (*p* = 0.014), miR-503-5p (*p* = 0.003), miR-365a-3p (*p* = 0.02), miR-34a-5p (*p* < 0.001), and miR-34a-3p (*p* = 0.006)] (Figures 2A–E) and overall aGvHD clinical grade [let-7c-5p (*p* = 0.005), miR-503-5p (*p* = 0.02), miR-365a-3p (*p* = 0.01), miR-34a-5p (*p* < 0.001), and miR-34a-3p (*p* = 0.003)] (Figures 2F–J). miR-142-3p, miR-21-3p, miR-24-3p, and miR-23b-3p did not retain their significance (*p* < 0.05). Focusing on the histopathological skin aGvHD stage, there was a significant down-regulation of let-7c-5p expression in the pre-transplant biopsies in comparison to the healthy controls (*p* = 0.04) (Figure 2A). miR-503-5p expression was upregulated in skin biopsies taken from patients with skin aGvHD stages 2–3 in comparison to the pre-transplant group (*p* = 0.032) (Figure 2B). miR-365a-3p expression levels were downregulated in biopsies taken from patients with stages 0–1 skin aGvHD compared with the pre-transplant (Tx) cohort (Figure 2C). miR-34a-5p expression levels were significantly up-regulated post-allo-HSCT, regardless of skin aGvHD stage (pre-Tx versus 0–1 *p* = 0.004, pre-Tx versus 2–3 *p* = 0.002) (Figure 2D). In addition, the expression of miR-34a-5p and miR-34a-3p was significantly positively correlated in skin biopsies in this cohort (*r*_s_ = 0.50, *p* = 0.006) (Figure S2 in Supplementary Material).

### Lower Cutaneous miR-503-5p and miR-34a-3p Expression Is Associated With Improved OS

**Figure 3:**
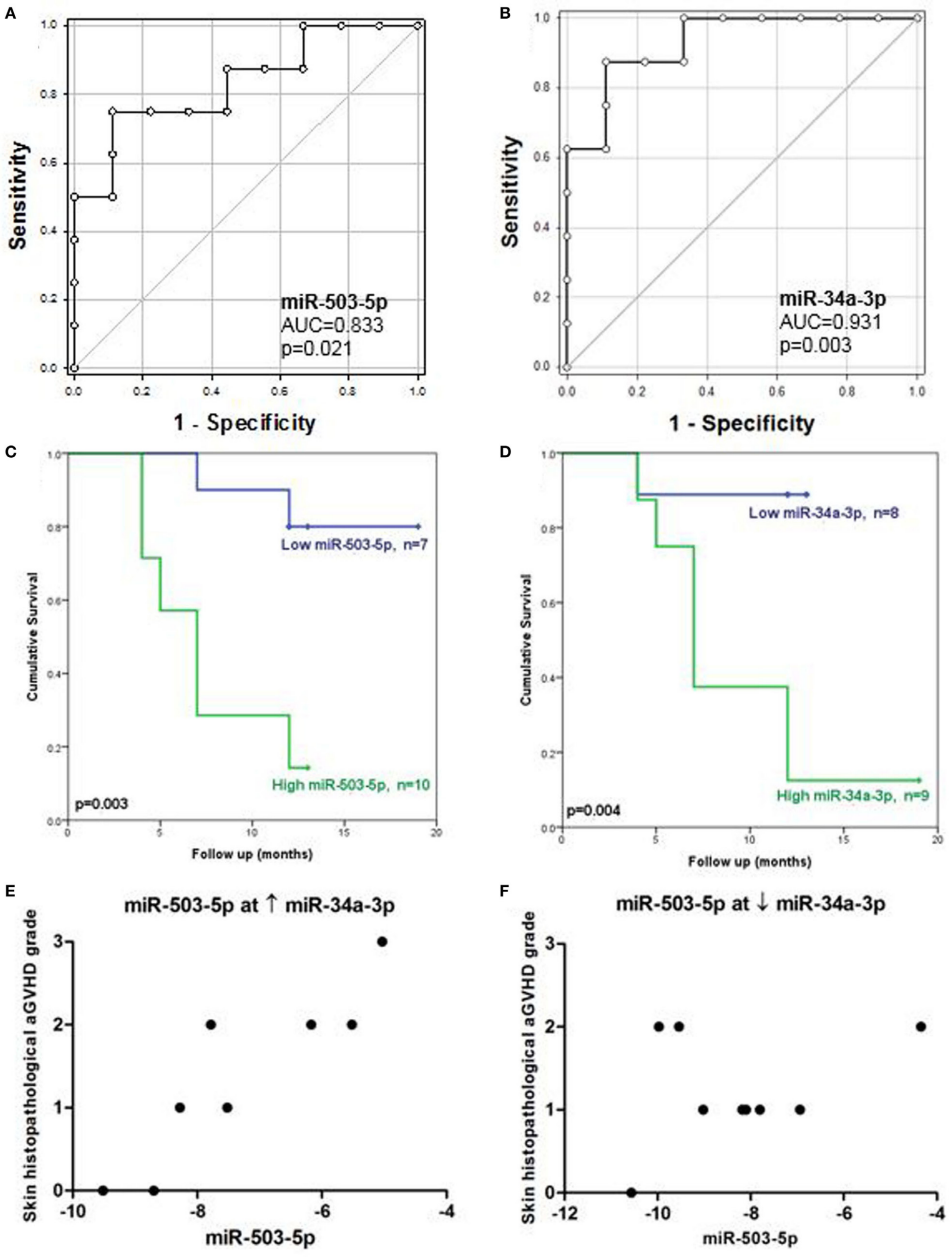
Association between cutaneous miR-503-5p and miR-34a-3p expression with overall survival (OS) and skin acute graft-versus-host disease (aGVHD) severity in the validation cohort. **(A–D)** Expression of miR-503-5p and miR-34a-3p in the skin validation cohort (*n* = 17) was analyzed according to OS. **(A,B)** miR-503-5p and miR-34a-3p expression was associated with OS according to receiver operator characteristic (ROC) analysis. ROC curves detailing area under the curve (AUC) and *p*-value significance are shown. **(C,D)** miR-503-5p and miR-34a-3p expression was dichotomized (low or high expression), based on the optimal thresholds evaluated by ROC analysis. Kaplan–Meier survival curves for OS were then generated, based on dichotomized microRNA expression. *p*-Values were calculated using the Log-Rank test. For both microRNAs, high expression was significantly associated with poor OS. **(E)** In patients with high levels of miR-34a-3p, miR-503-5p expression was associated with skin histopathological aGvHD. **(F)** However, in patients with low levels of miR-34a-3p, there was no association between miR-503-5p expression and skin aGvHD severity. **(E,F)** Histopathological grades are shown on the *y*-axis. The *x*-axis denotes microRNA expression. miR-503-5p expression levels were dichotomized based on miR-34a-3p expression.

Receiver operating characteristic analysis of the validation cohort, based on individual microRNA expression, showed that miR-503-5p and miR-34a-3p predicted for OS (miR-503-5p AUC = 0.83, *p* = 0.021, miR-34a-3p AUC = 0.93, *p* = 0.003) (Figures 3A,B). MicroRNA expression was dichotomized (low or high expression), based on the optimal thresholds evaluated by ROC analysis. The K.M method was used to generate survival plots. Low expression of miR-503-5p and miR-34a-3p was significantly associated with longer OS [miR-503-5p 17.1 versus 7.4 months median survival, L.R *p* = 0.003, hazard ratio (HR) = 7.7 (*p* = 0.014) and miR-34a-3p 12.0 versus 9.1 months median survival, L.R *p* = 0.004, HR = 10.93 (*p* = 0.03)] (Figures 3C,D).

To obtain a set of predictors in a multivariate model for OS, variable selection was performed using both the clinical risk factors and microRNAs. From the preliminary univariate variable selection (which included patient age, donor-patient relationship, patient gender, CMV status, graft regimen, skin aGvHD stage, mismatches in HLA Class I, and underlying disease), clinical risk factors with *p* < 0.2 (24) were chosen as candidates in a “Forward Likelihood Ratio” stepwise Cox regression procedure alongside the microRNAs [i.e., patient CMV status (*p* = 0.146), mismatches in HLA Class I (*p* = 0.187), skin histopathological aGvHD stage (*p* = 0.115), miR-503-5p (*p* = 0.014), and miR-34a-3p (*p* = 0.026) expression] (Table S3 in Supplementary Material). After variable selection, miR-503-5p [*p* = 0.020, HR = 6.87 (95% CI: 1.35–34.89)] was identified as an independent factor in the Cox regression equation.

### Statistical Interaction Between Cutaneous miR-503-5p and miR-34a-3p Is Associated With Skin aGvHD Stage

Statistical interactions between all microRNAs were also assessed in the validation cohort. Ordinal logistic regression analysis of skin histopathological aGvHD stage was performed incorporating both the main effect and interaction of all the validated microRNAs in a stepwise manner. Results showed that there was a statistically significant interaction between miR-503-5p and miR-34a-3p expression (*p* = 0.016), with miR-503-5p having the most significant effect (*p* = 0.011) and miR-34a-3p as the moderator (*p* = 0.020) in this interaction. Clinical covariates (as for the survival analysis) were considered in the stepwise procedure, but none were significant in the final model.

A conditional relationship was also identified between miR-503-5p and miR-34a-3p, such that when high levels of miR-34a-3p were expressed, higher levels of miR-503-5p were more clearly associated with severity of skin histopathological aGvHD stage (Figure 3E). However, the relationship between miR-503-5p and skin histopathological aGvHD severity was less apparent when levels of miR-34a-3p expression were low (Figure 3F). This finding requires further investigation to elucidate whether a clinical or biological factor was the reason for this trend. To further understand the conditional interaction between miR-503-5p and miR-34a-3p, rotational 3D scatterplots were generated (Figure S3 in Supplementary Material). Since miR-503-5p and miR-34a-3p were significantly associated with OS, non-parametric density contours based on the patient survival status were included, to aid visualization. The plots demonstrated the interaction between miR-503-5p and miR-34a-3p expression. None of the other microRNAs were associated with skin aGvHD stage, OS, or relapse.

### c-Myc and p53-Positive Cells Are Present in Cutaneous Biopsies of Allo-HSCT Patients

**Figure 4:**
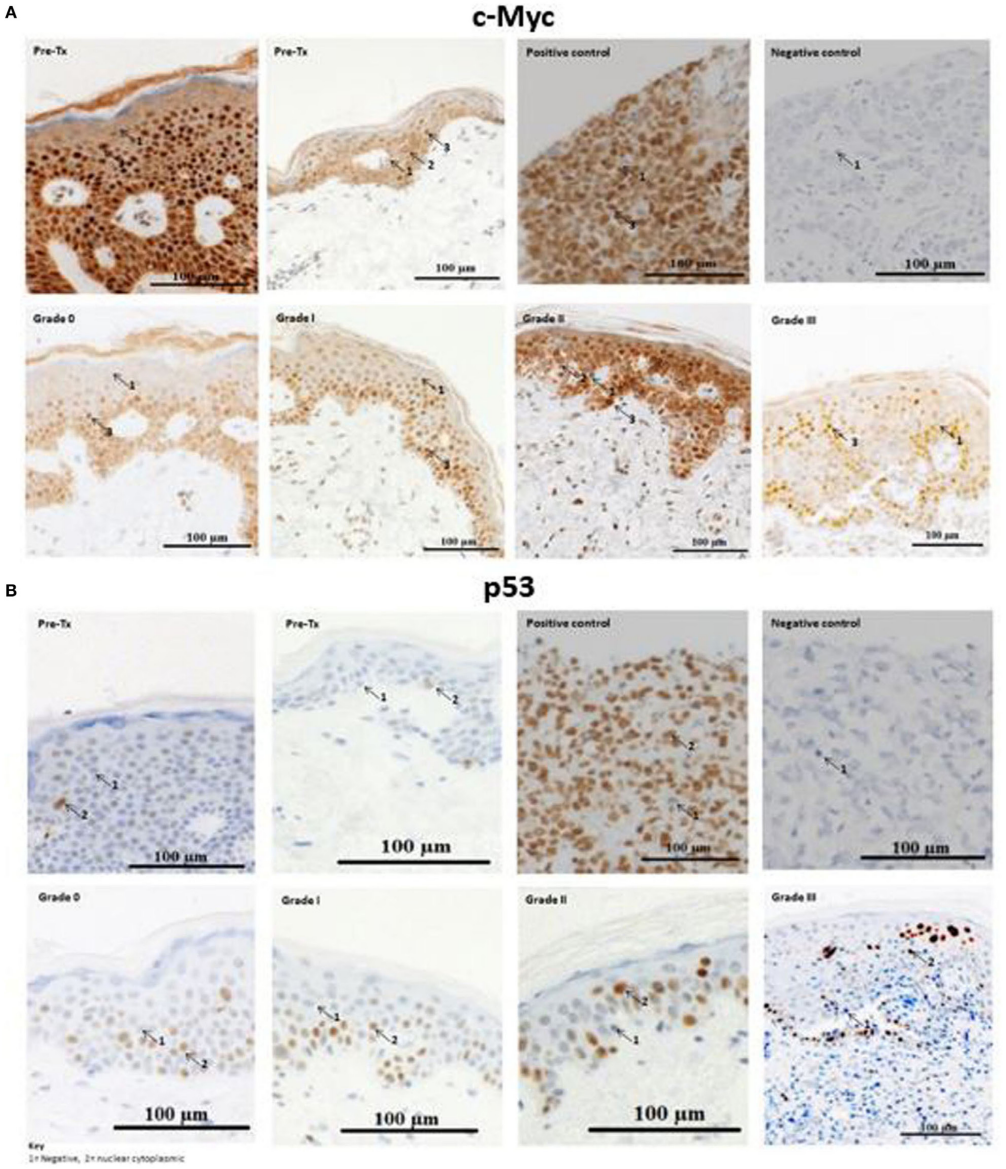
Example skin biopsy positivity for c-Myc and p53 protein. **(A)** c-Myc-positive cells demonstrated variable expression intensities (low, moderate, and high). No skin acute graft-versus-host disease (aGvHD) biopsies had high intensity c-Myc-positive cells in the basement membrane, while in stages 2–3 skin aGvHD positivity for c-Myc was observed throughout the entire epidermis. Positive-c-Myc proteins were also present in the pre-transplant skin biopsies. **(B)** p53-positive cells were present in pre-transplant skin biopsies, but were limited in number. Strong positive staining was observed with skin aGvHD severity. **(A,B)** The first row shows the two pre-transplant skin sections that were taken from the individuals with cutaneous histopathological aGvHD stage 0 and stage 1 (second row), respectively. Stage 3 skin sections showed severe cutaneous aGvHD, with separation between the epidermis and dermis. For both c-Myc and p53, the number of cells that infiltrated the dermis gradually increased from no GVHD (stage 0) to severe skin aGvHD (stage 3). Breast carcinoma tissues were used as a negative and positive control for c-Myc and p53. The images were taken at 20× magnification using AxioCamMR3.

Immunohistochemical analysis was performed to test whether c-Myc and p53 proteins, both of which are miR-34a-5p targets, were expressed in cutaneous biopsies from allo-HSCT patients pre- and post-transplantation. The majority of the activated c-Myc-positive cells, particularly those with high c-Myc intensity, were present in the basement membrane of skin histopathological stage 0 and stage 1 aGvHD biopsies (*n* = 9) (Figure 4). In stage 2 and stage 3 aGvHD biopsies (*n* = 7), the complete epidermis stained positive for c-Myc and very few negatively staining cells were present. A limited number of cells demonstrated cytoplasmic-only staining for c-Myc, while the majority showed both cytoplasmic and nuclear staining.

Likewise, cells that positively stained for p53 protein (Figure 4) were also analyzed for differences between pre- and post-allo-HSCT skin biopsies. Visualization of the sections showed positive cytoplasmic-nuclear staining for p53 under all histopathological conditions (pre-transplantation to stage 3 aGvHD). p53-positive cells were localized in the epidermis. In healthy volunteers, p53-positive cells are confined to the basement membrane (25). Only occasional cells in the dermis were p53-positive. The majority of p53-positive cells stained with strong to moderate intensity. The dermis had less than 4% p53-positive cells and was therefore considered as p53-negative. Thus, for p53, the quick score method was calculated based only on the epidermis.

### p53 Positively Correlates With miR-34a-5p Expression in the Epidermis

There was no statistically significant correlation between miR-34a (-5p or -3p) expression levels and the proportion of c-Myc-positive cells (*r*_s_ = 0, *p* > 0.05). No association with the percentage of c-Myc-positive cells was identified, and this was confirmed by performing Jonckheere’s trend test (*p* = 0.988) (data not shown).

Interestingly, in all samples (*n* = 22), comprising both the validation cohort (*n* = 17) and pre-transplant biopsies (*n* = 5), miR-34a-5p expression alone positively correlated with the percentage of p53-positive cells in the epidermis (*r*_s_ = 0.44, *p* = 0.039) (Figure S4 in Supplementary Material). In post-allo-HSCT skin biopsies only (*n* = 17), there was no significant correlation between p53-positive cells and microRNA expression. There was a significant difference between the percentage p53-positive cells pre-transplantation and skin aGvHD stages 2 and 3 (*p* = 0.045) (Figure S4 in Supplementary Material). Jonckheere’s trend test confirmed the significant trend toward higher p53-positive cells with increasing skin aGvHD stage post allo-HSCT (*p* = 0.009).

### Serum Expression of miR-503-5p, miR-34a-3p, miR-34a-5p, and let-7c-5p Is Associated With aGvHD Incidence

**Figure 5:**
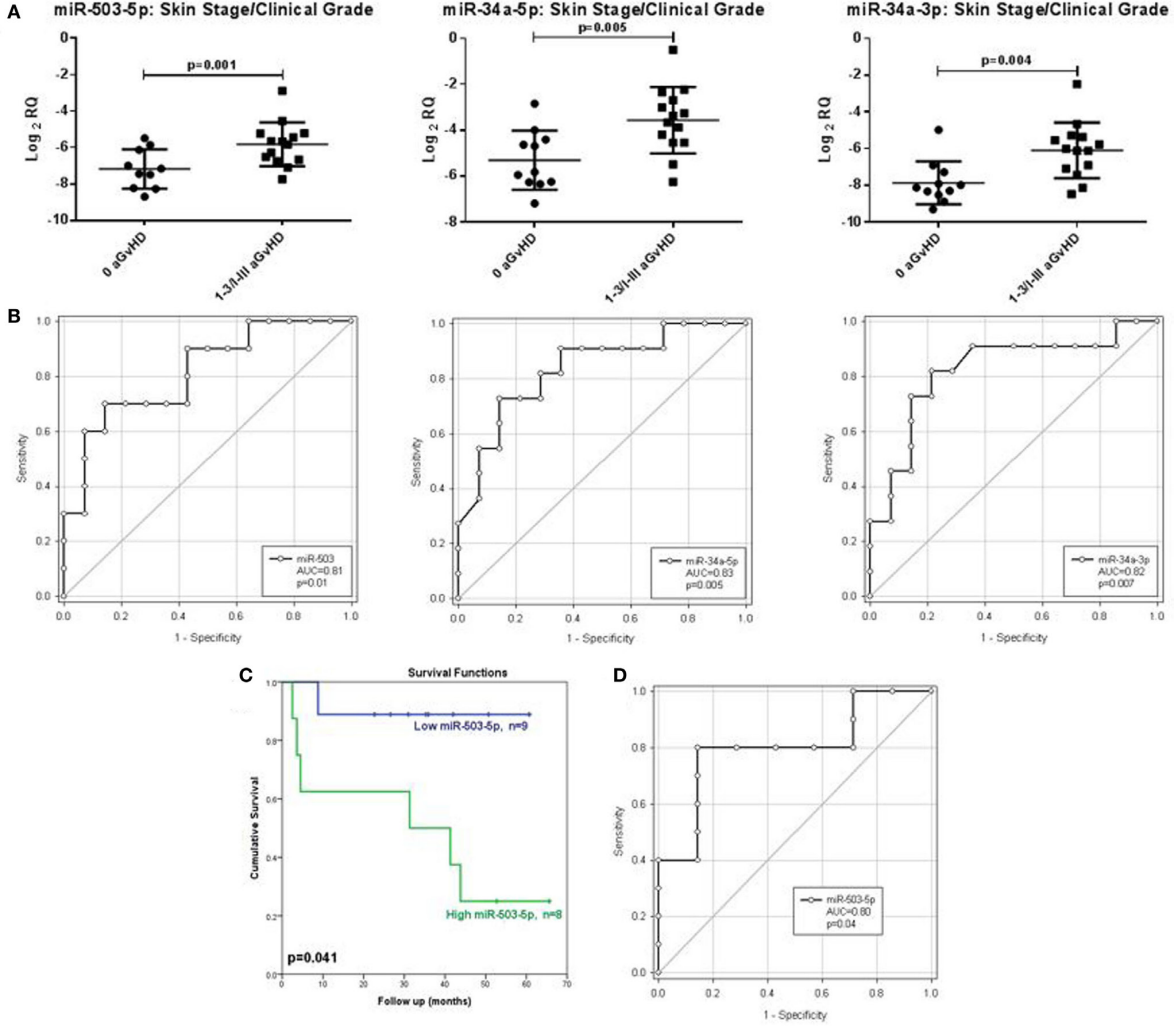
Association between serum expression of miR-503-5p, miR-34a-5p and miR-34a-3p with skin histopathological aGvHD incidence, overall clinical aGvHD grade and overall survival. **(A)** Scatterplots demonstrating miR-503-5p, miR-34a-5p, and miR-34a-3p expression (mean and SEM) in serum samples at Day 28 post-HSCT according to skin aGvHD stage/overall aGvHD grade. Significance was determined using the Student’s *t*-test. **(B)** Receiver operator characteristic (ROC) curves detailing area under the curve (AUC) and *p*-value significance for miR-503-5p, miR-34a-5p, and miR-34a-3p with incidence of skin aGvHD/clinical aGvHD. **(C)** Kaplan–Meier survival curves for OS, based on dichotomized miR-503-5p expression. *p*-Values were calculated using the Log-Rank test. **(D)** ROC curve detailing AUC and *p*-value significance for miR-503-5p and OS.

In order to assess the predictive capacity of circulatory microRNAs, expression of miR-503-5p, miR-34a-5p, miR-34a-3p, and let-7c-5p was further investigated at day 28 (D28) post-HSCT in transplant patient serum samples (*n* = 30) (Table 3). Analyses were based on both skin histopathological stage (mean onset 49 days post-HSCT, stage 0 *n* = 13, stage 1 *n* = 10, stage 2 *n* = 4, stage 3 *n* = 3) and overall clinical aGvHD grade (mean onset 54 days post-HSCT, grade 0 *n* = 13, grade I *n* = 9, grade II *n* = 5, grade III *n* = 3). Initial analysis indicated that expression of all microRNAs was higher in patients with both skin and overall clinical aGvHD, including milder stage/grade 1/I, compared with no-aGvHD (data not shown). Thus, patient groups were dichotomized according to skin stage 0 versus 1–3 or overall grade 0 versus I–III for subsequent analysis. miR-503-5p (*p* = 0.001), miR-34a-5p (*p* = 0.005), and miR-34a-3p (*p* = 0.004) were expressed at a higher level in patients at D28 post-HSCT who developed both skin and overall aGvHD (stages 1–3, grades I–III) (*n* = 17), compared with patients with no skin or overall aGvHD (*n* = 13) (Figure 5A).

When microRNAs were assessed in relation to individual skin aGvHD stage or clinical aGvHD grade, there was no significant difference in expression between disease stages or grades (*p* > 0.05, data not shown), indicating the microRNAs to be associated with skin and overall aGvHD incidence, but not severity (data not shown).

Following identification of differential expression of candidate microRNAs in patients who developed skin and overall aGvHD, ROC analysis was performed to identify time point-specific microRNA prognostic ability. ROC analysis demonstrated miR-503-5p (*p* = 0.01, AUC = 0.81), 34a-5p (*p* = 0.005, AUC = 0.83), and miR-34a-3p (*p* = 0.007, AUC = 0.82) to have prognostic ability at D28 with regard to aGvHD incidence (Figure 5B).

Correlation analysis was performed between all microRNAs, in both aGvHD and no-aGvHD patients. For aGvHD patients, correlation was detected between expression of miR-34a-5p and miR-34a-3p (*r* = 0.88, *p* < 0.0001) and, miR-34a-5p and miR-503-5p (*r* = 0.51, *p* = 0.03), but not between miR-34a-3p and miR-503-5p (*r* = 0.35, *p* = 0.14). When assessing patients who did not develop aGvHD, no correlation was observed between expressions of any of the microRNAs (*p* > 0.05).

### Lower Serum miR-503-5p Expression Is Associated With Improved OS

Cox regression analysis showed no significant relation between OS and miR-34a-5p (HR = 0.914, *p* = 0.61) nor miR-34a-3p (HR = 0.97, *p* = 0.86) serum expression at D28. Expression of miR-503-5p, however, showed a significant association with OS (HR = 6.86, *p* = 0.038).

miR-503-5p expression was dichotomized (low or high expression), based on the optimal thresholds evaluated by ROC analysis, and K.M plots were used to analyze cumulative survival. High miR-503-5p levels at D28 were significantly associated with poor OS (32.3 versus 54.9 months median survival, L.R *p* = 0.041) (Figure 5C). ROC analysis demonstrated miR-503-5p at day 28 (*p* = 0.04, AUC = 0.80) to have prognostic ability with regard to OS (Figure 5D).

## Discussion

The critical role of microRNA in disease pathology and development has been highlighted in numerous studies to-date (26). MicroRNAs are cell-specific, stable, and therefore ideal biomarkers (27). Thus, this study aimed to identify a signature list of microRNAs that could discriminate mild (stage 1) from moderate to severe (stages 2 and 3) cutaneous aGvHD. Global microRNA profiling by RT-qPCR was carried out on normal and post-HSCT biopsies taken from patients with cutaneous aGvHD. Eight microRNAs that were differentially expressed across the groups (miR-142-3p, miR-34a-5p, miR-503-5p, let-7c-5p, miR-21-3p, miR-365a-3p, miR-23b-3p, and miR-24-3p) were selected for individual validation by RT-qPCR in healthy controls and pre-transplant and post-transplant skin biopsies from patients with and without aGvHD. Four microRNAs (miR-34a-5p, miR-34a-3p, let-7c-5p, and miR-503-5p) were further investigated in serum samples for their circulatory biomarker potential for skin and overall aGvHD.

Unsupervised hierarchical clustering of the global microRNA expression levels showed that there were two distinct clusters, supporting the hypothesis that healthy controls and allo-HSCT patients have different cutaneous microRNA expression patterns. The findings suggested that microRNAs could be deregulated as a result of both the allo-HSCT procedure and aGvHD disease onset. This is in agreement with previous studies that have reported differential expression of circulatory microRNAs post-cytotoxic therapy prior to HSCT, as well as in relation to aGvHD onset (10, 28). In the subsequent validation study, five microRNAs of the eight observed in the discovery cohort retained their significance (let-7c-5p, miR-503-5p, miR-365a-3p, and miR-34a-5p/-3p). Interestingly, only let-7c was significantly differentially expressed between normal controls and pre-HSCT patients (lower in pre-HSCT), suggesting expression of this microRNA to be altered as a results of underlying disease. The results showed that let-7c-5p expression increased after transplantation, but it was surprising to find that levels in skin biopsies with skin aGvHD histopathological stages 2–3 were approximately the same as that of healthy controls. A previous study demonstrated that let-7c-5p is highly expressed in CD4^+^ T-cells in healthy individuals (29). Results in the healthy controls were, therefore, in concordance with this study. Swaminathan et al. (29) have shown that the expression of IL-10 protein could be modulated by let-7c-5p. IL-10 is an anti-inflammatory cytokine that has been extensively studied in GvHD (30). At this stage, it could be postulated that let-7c-5p might play a role in immune-regulation rather than cutaneous aGvHD.

As the skin is involved in ~80% of GvHD cases, further understanding the repertoire of microRNA expression in a cutaneous aGvHD setting is important in order to better understand the complex nature of this disease. Although the data presented here strengthens the potential of miR-503-5p, miR-34a-5p, miR-34a-3p, and let-7c-5p for use as prospective cutaneous aGvHD biomarkers, the necessity for invasive biopsies is not advantageous. Therefore, studies were extended to assess their expression in serum samples pre- and post-transplant. Results showed that miR-503-5p, miR-34a-5p, and miR-34a-3p retained their biomarker potential with respect to skin and overall clinical aGvHD incidence, as demonstrated by high expression in aGvHD patients at D28 post-HSCT and subsequent ROC analysis.

When microRNA expression was assessed in relation to post-HSCT survival, lower miR-503-5p expression levels were associated with improved OS when assessed in both the skin and the serum. miR-503 has been shown to negatively regulate CD40 gene expression when malignant human monocyte-derived cells (U937 cells) were irradiated (31). The function of the CD40-CD40 ligand (CD40L) pathway has been investigated in GvHD models (32). T and NK cells are activated when CD40 and CD40L ligate, which results in the release of established pro-inflammatory cytokines such as IFN-γ, IL-2, and IL-12 (33). Studies have shown that anti-CD40L antibodies can decrease GvHD severity (32). Activated CD4^+^ T-cells express CD40L that can be targeted by anti-CD40L antibodies. These antibodies block CD4^+^ T-cells, thus making them tolerant to host antigens, resulting in lower GvHD (34). Moreover, CD40 is also expressed in human keratinocytes in the skin (35). Since miR-503 and miR-424 are polycistronic, they have very similar seed sequences and have been shown to target the same genes and also be involved in monocyte to macrophage differentiation in an AML cell line study (36). In a GvHD-related study, investigators have shown that miR-424 levels are higher in cord blood CD4^+^ and CD8^+^ cells when compared to adult peripheral blood cells (37). It was also demonstrated that miR-424 is overexpressed in adult blood CD14^+^ monocytes, when compared with CD4^+^ and CD8^+^ T-cells (37). It is known that during inflammation monocytes are recruited to the affected site and differentiate to either macrophages and/or DCs [as reviewed in Ref. (38)]. Therefore, miR-503-5p could be overexpressed to target CD40 gene expression and thus, inhibit T-cell activation. Its expression levels may also be upregulated as more monocytes are recruited and migrate from the blood to the host skin tissue.

Likewise, miR-365a-3p was under-expressed post-transplantation. Xu et al. (39) have shown that miR-365a-3p is a negative regulator of IL-6, which is a cytokine involved in the control of immune responses (40). Studies have demonstrated that IL-6 levels are elevated at the time of aGvHD onset in the sera of patients (41). Skin fibroblasts have also been shown to express IL-6 under inflammatory conditions (42). It may be possible that the decrease in miR-365a-3p expression post-transplant causes IL-6 level to increase in aGvHD patients.

miR-34a-5p was significantly differentially expressed between pre-transplant and skin stages 0–1 as well as stages 2–3 aGvHD biopsies. Similarly, it was differentially expressed between pre-transplant and grades 0–I and grades II–IV aGvHD when assessed according to overall clinical aGvHD. Expression was also significantly higher in D28 serum samples in patients who developed overall aGvHD or skin aGvHD. However, no significant difference was observed between mild and moderate aGvHD in either the skin or the serum. Interestingly, miR-34a was not expressed at all when tested in a small cohort of scleroderma skin biopsies (*n* = 5, data not shown). Thus, the findings suggest that miR-34a-5p may be upregulated as a result of the allo-HSCT, as well as inflammation due to GvHD. miR-34a has previously been associated with aGvHD in Fanconi anemia (FA) (6). Patients with FA are particularly susceptible to developing high-grade aGvHD, thought to be mediated through impaired DNA repair processes leading to increased apoptosis (43). It was demonstrated that miR-34a expression was higher in aGvHD compared with no-aGvHD, in both the skin and gut and that expression directly correlated with the number of apoptotic cells present in the gut (6). This supports the findings of this study in which miR-34a levels were elevated in aGvHD. Thus, serum miR-34a levels may be indicative of apoptosis occurring in target GvHD organs, thus serving as a potential serum biomarker of aGvHD. In allo-HSCT and GvHD, T-cell receptors (TCRs) are stimulated during the GvH cycle when T-cells recognize HLA mismatches between the patient and donor. This results in partial activation of the T-cells (44). Recently, it has been shown that overexpression of miR-34a is related to higher TCR T-cell activation. The positive correlation between miR-34a expression and TCR stimulation has been linked to diacylglycerol kinase-zeta (DAGK-ζ), which is an enzyme responsible for metabolizing diacylglycerol (DAG) (45). Lower DAGK-ζ results in higher T-cell activation as the cells become sensitive to TCR triggers, and miR-34a directly targets DAGK-ζ mRNA, leading to more un-metabolized DAG, which stimulates TCRs and enhanced T-cell activation (45).

In this study, low expression levels of miR-503-5p and miR-34a-3p were indicative of improved OS. Interestingly, when considering skin histopathological aGvHD stage as the outcome, a significant statistical interaction was present between miR-503-5p and miR-34a-3p and this was distinctly evident when survival status was added onto 3D plots for visualization. The biological mechanism underlying the statistical interaction needs further investigation, but our results are consistent with a “gate” mechanism, whereby at high levels of miR-34a-3p, miR-503-5p is the dominant effector and most patients have a lower chance of survival, while higher levels of miR-503-5p accompany more severe aGvHD. By contrast, as miR-34a-3p expression decreased, the effect of miR-503-5p was negligible, most patients survived, and the severity of aGvHD increased. It would be interesting to assess whether cutaneous microRNA expression patterns directly reflect aGvHD severity. However, patients who develop severe aGvHD are rare in the Newcastle Transplantation facility due to the controlled conditioning and prophylaxis treatments. Almost every patient in this center is administered with alemtuzumab (Campath). In this study, only one patient from the cutaneous cohort was diagnosed with skin aGvHD stage 3. Therefore, we could only analyze stage 2 and 1 versus pre-transplant biopsies. None of the microRNAs were associated with risk of relapse and it was not possible to perform competing risk analysis, due to the few cases present per group. It will therefore be important to validate these results in larger, multi-center studies that incorporate a higher number of high stage 3-4 aGvHD patients. Furthermore, although it is encouraging that the potential biomarkers identified in this study can be confirmed in an independent cohort, additional expanded cohorts will allow for sub-stratification in order to investigate the impact of heterogeneous conditioning regimens and pre-transplant prophylaxis, as well as improve the strength of multivariate analyses.

In this study, whole skin biopsies were used for microRNA expression analysis. However, aGvHD mainly attacks the epidermal layer of the skin (2). Thus, the microRNA expression profiles observed are collective and may decrease the discriminatory specificity of the results. In addition, the proportion and types of cells in the epidermis and dermis are different. Thus, in order to fully understand the biology of the microRNAs, it may be essential to assess their expression levels separately in each layer of the skin, separated by laser capture microdissection.

The protein expression of microRNA targets was investigated in the same cohort. Results showed that c-Myc and p53-positive cells were present in cutaneous biopsies of allo-HSCT patients. The proto-oncogene c-Myc is involved in fundamental processes such as differentiation, apoptosis, and cellular proliferation (46, 47). In this study, no significant association was determined between miR-34a expression and c-Myc-positivity; however, significant positive correlation between p53-positive cells and miR-34a-5p expression was observed. This result is in support of the p53-positive feedback loop with miR-34a (12). p53-positive cells were observed even in pre-transplantation skin biopsies, and overall the number of p53-positive cells increased with aGvHD severity (*p* = 0.045). Taken together, these results may suggest that pre-transplant activation of p53 is due to the conditioning regimen, which is not sufficient to trigger miR-34a-5p overexpression. In the post allo-HSCT skin, p53 is overexpressed due to the conditioning regimen as well as the GvH reaction, which led to higher miR-34a-5p expression and therefore positive correlation in their expression.

In summary, this research has shown that miR-34a, miR-503-5p, let-7c-5p, and miR-365a-3p may be implicated in both the transplant procedure and the pathogenesis of aGvHD. Expression of miR-503-5p, miR-34a-5p, and miR-34a-3p in serum demonstrated potential non-invasive biomarker potential. Further investigation into the experimentally validated targets of miR-34a, miR-503-5p, let-7c-5p, and miR-365a-3p may shed light on their impact at the protein level and their roles in maintaining skin homeostasis. These results need to be validated in a larger, multicentre cohort.

## Ethics Statement

Research was granted ethical approval by the Newcastle and North Tyneside Research Ethics Committee. Participants gave full informed written consent for their samples to be used for research purposes.

## Author Contributions

SA designed and performed the experiments, evaluated the data, and helped prepare the manuscript. JN assisted with designing and performing experiments and drafting of the manuscript. LB performed experiments in serum samples. AJ, PR, and X-NW graded the patients GvHD and non-GvHD skin sections. CL performed clinical data collection and advised on statistical interactions analysis. KP advised and performed statistical analysis. SO and JL provided scleroderma samples. MC contributed to clinical samples and data collection. RC and AD developed the overall concept, supervised the research, and prepared the manuscript. All the authors approved the final manuscript.

## Conflict of Interest Statement

The authors declare that the research was conducted in the absence of any commercial or financial relationships that could be construed as a potential conflict of interest.

## References

[1] Ziemer M. Graft-versus-host disease of the skin and adjacent mucous membranes. J Dtsch Dermatol Ges (2013) 11:477–95. 10.1111/ddg.12103 23721594

[2] Hofmeister CC Quinn A Cooke KR Stiff P Nickoloff B Ferrara JL. Graft-versus-host disease of the skin: life and death on the epidermal edge. Biol Blood Marrow Transplant (2004) 10:366–72. 10.1016/j.bbmt.2004.03.003 15148490

[3] Glucksberg H Storb R Fefer A Buckner CD Neiman PE Clift RA Clinical manifestations of graft-versus-host disease in human recipients of marrow from HL-A-matched sibling donors. Transplantation (1974) 18:295–304. 10.1097/00007890-197410000-00001 4153799

[4] Stickel N Prinz G Pfeifer D Hasselblatt P Schmitt-Graeff A Follo M miR-146a regulates the TRAF6/TNF-axis in donor T cells during GvHD. Blood (2014) 124(16):2586–95. 10.1182/blood-2014-04-569046 25205119

[5] Ranganathan P Heaphy CE Costinean S Stauffer N Na C Hamadani M Regulation of acute graft-versus-host disease by microRNA-155. Blood (2012) 119:4786–97. 10.1182/blood-2011-10-387522 22408260 PMC3367879

[6] Wang L Romero M Ratajczak P Leboeuf C Belhadj S Peffault De Latour R Increased apoptosis is linked to severe acute GVHD in patients with Fanconi anemia. Bone Marrow Transplant (2013) 48:849–53. 10.1038/bmt.2012.237 23222379

[7] Leonhardt F Grundmann S Behe M Bluhm F Dumont RA Braun F Inflammatory neovascularization during graft-versus-host disease is regulated by alphav integrin and miR-100. Blood (2013) 121:3307–18. 10.1182/blood-2012-07-442665 23327924

[8] Xiao B Wang Y Li W Baker M Guo J Corbet K Plasma microRNA signature as a noninvasive biomarker for acute graft-versus-host disease. Blood (2013) 122:3365–75. 10.1182/blood-2013-06-510586 24041574 PMC3821726

[9] Crossland RE Norden J Kralj Juric M Pearce KF Lendrem C Bibby LA Serum and extracellular vesicle microRNAs miR-423, miR-199, and miR-93* as biomarkers for acute graft-versus-host disease. Front Immunol (2017) 8:1446 10.3389/fimmu.2017.01446 29176973 PMC5686047

[10] Crossland RE Norden J Juric MK Green K Pearce KF Lendrem C Expression of serum microRNAs is altered during acute graft-versus-host disease. Front Immunol (2017) 8:308 10.3389/fimmu.2017.00308 28392786 PMC5364146

[11] Jalapothu D Boieri M Crossland RE Shah P Butt IA Norden J Tissue-specific expression patterns of microRNA during acute graft-versus-host disease in the rat. Front Immunol (2016) 7:361. 10.3389/fimmu.2016.00361 27695455 PMC5025478

[12] Yamakuchi M Lowenstein CJ. miR-34, SIRT1 and p53: the feedback loop. Cell Cycle (2009) 8:712–5. 10.4161/cc.8.5.7753 19221490

[13] Ebner OA Selbach M. Quantitative proteomic analysis of gene regulation by miR-34a and miR-34c. PLoS One (2014) 9:e92166. 10.1371/journal.pone.0092166 24637697 PMC3956911

[14] Wilson A Murphy MJ Oskarsson T Kaloulis K Bettess MD Oser GM c-Myc controls the balance between hematopoietic stem cell self-renewal and differentiation. Genes Dev (2004) 18:2747–63. 10.1101/gad.313104 15545632 PMC528895

[15] Jackstadt R Hermeking H. MicroRNAs as regulators and mediators of c-MYC function. Biochim Biophys Acta (2014).1849(5):544–53. 10.1016/j.bbagrm.2014.04.003 24727092

[16] Hahn S Jackstadt R Siemens H Hunten S Hermeking H. SNAIL and miR-34a feed-forward regulation of ZNF281/ZBP99 promotes epithelial-mesenchymal transition. EMBO J (2013) 32:3079–95. 10.1038/emboj.2013.236 24185900 PMC3844956

[17] Yada S Takamura N Inagaki-Ohara K O’leary MK Wasem C Brunner T The role of p53 and Fas in a model of acute murine graft-versus-host disease. J Immunol (2005) 174:1291–7. 10.4049/jimmunol.174.3.1291 15661885

[18] Andersen CL Jensen JL Orntoft TF. Normalization of real-time quantitative reverse transcription-PCR data: a model-based variance estimation approach to identify genes suited for normalization, applied to bladder and colon cancer data sets. Cancer Res (2004) 64:5245–50. 10.1158/0008-5472.CAN-04-0496 15289330

[19] Crossland RE Norden J Bibby LA Davis J Dickinson AM Evaluation of optimal extracellular vesicle small RNA isolation and qRT-PCR normalisation for serum and urine samples. J Immunol Methods (2016) 429:39–49. 10.1016/j.jim.2015.12.011 26723490

[20] Detre S Saclani Jotti G Dowsett M A “quickscore” method for immunohistochemical semiquantitation: validation for oestrogen receptor in breast carcinomas. J Clin Pathol (1995) 48:876–8. 10.1136/jcp.48.9.876 7490328 PMC502883

[21] Hinton PR Interaction of Factors in the Analysis of Variance. New York: Routledge (2014).

[22] Wang Y Zhang Z Ji D Chen G-F Feng X Gong L-L Regulation of T cell function by microRNA-720. Sci Rep (2015) 5:12159. 10.1038/srep12159 26199080 PMC4510490

[23] Rasmussen KD Simmini S Abreu-Goodger C Bartonicek N Di Giacomo M Bilbao-Cortes D The miR-144/451 locus is required for erythroid homeostasis. J Exp Med (2010) 207:1351–8. 10.1084/jem.20100458 20513743 PMC2901075

[24] Labopin M Iacobelli S Statistical Guidelines for EBMT [Online]. (2003). Available from: http://portal.ebmt.org/sites/clint2/clint/Documents/StatGuidelines_oct2003.pdf (Accessed: January, 2015).

[25] Baran W Szepietowski JC Szybejko-Machaj G Expression of p53 protein in psoriasis. Acta Dermatovenerol Alp Pannonica Adriat (2005) 14:79–83. 16200332

[26] Lu J Getz G Miska EA Alvarez-Saavedra E Lamb J Peck D MicroRNA expression profiles classify human cancers. Nature (2005) 435:834–8. 10.1038/nature03702 15944708

[27] Culpin RE Sieniawski M Proctor SJ Menon G Mainou-Fowler T. MicroRNAs are suitable for assessment as biomarkers from formalin-fixed paraffin-embedded tissue, and miR-24 represents an appropriate reference microRNA for diffuse large B-cell lymphoma studies. J Clin Pathol (2013) 66:249–52. 10.1136/jclinpath-2012-201021 23172553

[28] Walenda T Diener Y Jost E Morin-Kensicki E Goecke TW Bosio A MicroRNAs and metabolites in serum change after chemotherapy: impact on hematopoietic stem and progenitor cells. PLoS One (2015) 10:e0128231. 10.1371/journal.pone.0128231 26024523 PMC4449031

[29] Swaminathan S Suzuki K Seddiki N Kaplan W Cowley MJ Hood CL Differential regulation of the let-7 family of microRNAs in CD4+ T cells alters IL-10 expression. J Immunol (2011) 188:6238–46. 10.4049/jimmunol.1101196 22586040

[30] Lin M-T Storer B Martin PJ Tseng L-H Grogan B Chen P-J Genetic variation in the IL-10 pathway modulates severity of acute graft-versus-host disease following hematopoietic cell transplantation: synergism between IL-10 genotype of patient and IL-10 receptor β genotype of donor. Blood (2005) 106:3995–4001. 10.1182/blood-2004-11-4338 16109775 PMC1895107

[31] Cheng G Sun S Wang Z Jin S. Investigation of the interaction between the MIR-503 and CD40 genes in irradiated U937 cells. Radiat Oncol (2012) 7:38. 10.1186/1748-717X-7-38 22429276 PMC3325872

[32] Durie FH Aruffo A Ledbetter J Crassi KM Green WR Fast LD Antibody to the ligand of CD40, gp39, blocks the occurrence of the acute and chronic forms of graft-vs-host disease. J Clin Invest (1994) 94:1333–8. 10.1172/JCI117453 7521888 PMC295220

[33] Briones J Novelli S Sierra J T-cell costimulatory molecules in acute-graft-versus host disease: therapeutic implications. Bone Marrow Res (2011) 2011:976793 10.1155/2011/976793 22046574 PMC3195325

[34] Blazar BR Taylor PA Noelle RJ Vallera DA. CD4(+) T cells tolerized ex vivo to host alloantigen by anti-CD40 ligand (CD40L:CD154) antibody lose their graft-versus-host disease lethality capacity but retain nominal antigen responses. J Clin Invest (1998) 102:473–82. 10.1172/JCI3741 9691083 PMC508907

[35] Denfeld RW Hollenbaugh D Fehrenbach A Weiss JM Von Leoprechting A Mai B CD40 is functionally expressed on human keratinocytes. Eur J Immunol (1996) 26:2329–34. 10.1002/eji.1830261009 8898941

[36] Forrest AR Kanamori-Katayama M Tomaru Y Lassmann T Ninomiya N Takahashi Y Induction of microRNAs, MiR-155, MiR-222, MiR-424 and MiR-503, promotes monocytic differentiation through combinatorial regulation. Leukemia (2010) 24:460–6. 10.1038/leu.2009.246 19956200

[37] Takahashi N Nakaoka T Yamashita N. Profiling of immune-related microRNA expression in human cord blood and adult peripheral blood cells upon proinflammatory stimulation. Eur J Haematol (2012) 88:31–8. 10.1111/j.1600-0609.2011.01707.x 21913990

[38] Ginhoux F Jung S. Monocytes and macrophages: developmental pathways and tissue homeostasis. Nat Rev Immunol (2014) 14:392–404. 10.1038/nri3671 24854589

[39] Xu Z Xiao S-B Xu P Xie Q Cao L Wang D miR-365, a novel negative regulator of interleukin-6 gene expression, is cooperatively regulated by Sp1 and NF-𝝹B. J Biol Chem (2011) 286:21401–12. 10.1074/jbc.M110.198630 21518763 PMC3122200

[40] Akira S Hirano T Taga T Kishimoto T. Biology of multifunctional cytokines: IL 6 and related molecules (IL 1 and TNF). FASEB J (1990) 4:2860–7. 10.1096/fasebj.4.11.2199284 2199284

[41] Symington FW Symington BE Liu PY Viguet H Santhanam U Sehgal PB. The relationship of serum IL-6 levels to acute graft-versus-host disease and hepatorenal disease after human bone marrow transplantation. Transplantation (1992) 54:457–62. 10.1097/00007890-199209000-00014 1412727

[42] Koch AE Kronfeld-Harrington LB Szekanecz Z Cho MM Haines GK Harlow LA In situ expression of cytokines and cellular adhesion molecules in the skin of patients with systemic sclerosis. Their role in early and late disease. Pathobiology (1993) 61:239–46. 10.1159/000163802 7507681

[43] Guardiola P Socie G Li X Ribaud P Devergie A Esperou H Acute graft-versus-host disease in patients with Fanconi anemia or acquired aplastic anemia undergoing bone marrow transplantation from HLA-identical sibling donors: risk factors and influence on outcome. Blood (2004) 103:73–7. 10.1182/blood-2003-06-2146 12946993

[44] Baxter AG Hodgkin PD. Activation rules: the two-signal theories of immune activation. Nat Rev Immunol (2002) 2:439–46. 10.1038/nri823 12093010

[45] Shin J Xie D Zhong X-P. MicroRNA-34a enhances T cell activation by targeting diacylglycerol kinase ζ. PLoS One (2013) 8:e77983. 10.1371/journal.pone.0077983 24147106 PMC3798301

[46] Schuhmacher M Staege MS Pajic A Polack A Weidle UH Bornkamm GW Control of cell growth by c-Myc in the absence of cell division. Curr Biol (1999) 9:1255–8. 10.1016/S0960-9822(99)80507-7 10556095

[47] Pelengaris S Khan M Evan GI Suppression of Myc-induced apoptosis in β cells exposes multiple oncogenic properties of Myc and triggers carcinogenic progression. Cell (2002) 109:321–34. 10.1016/S0092-8674(02)00738-9 12015982

[48] Atarod S MicroRNAs in Haematopoietic Stem Cell Transplantation Outcome. Newcastle upon Tyne: Newcastle University (2015).

