# Supplementary material for: Differential MicroRNA Expression Levels in Cutaneous Acute Graft-Versus-Host Disease
Source: Front Immunol. 2018 Jul 10;9:1485. doi: 10.3389/fimmu.2018.01485 (PMC6048189; doi:10.3389/fimmu.2018.01485)
Supplement: Supplementary file 1 [file presentation_1.pptx]

## Slide 1
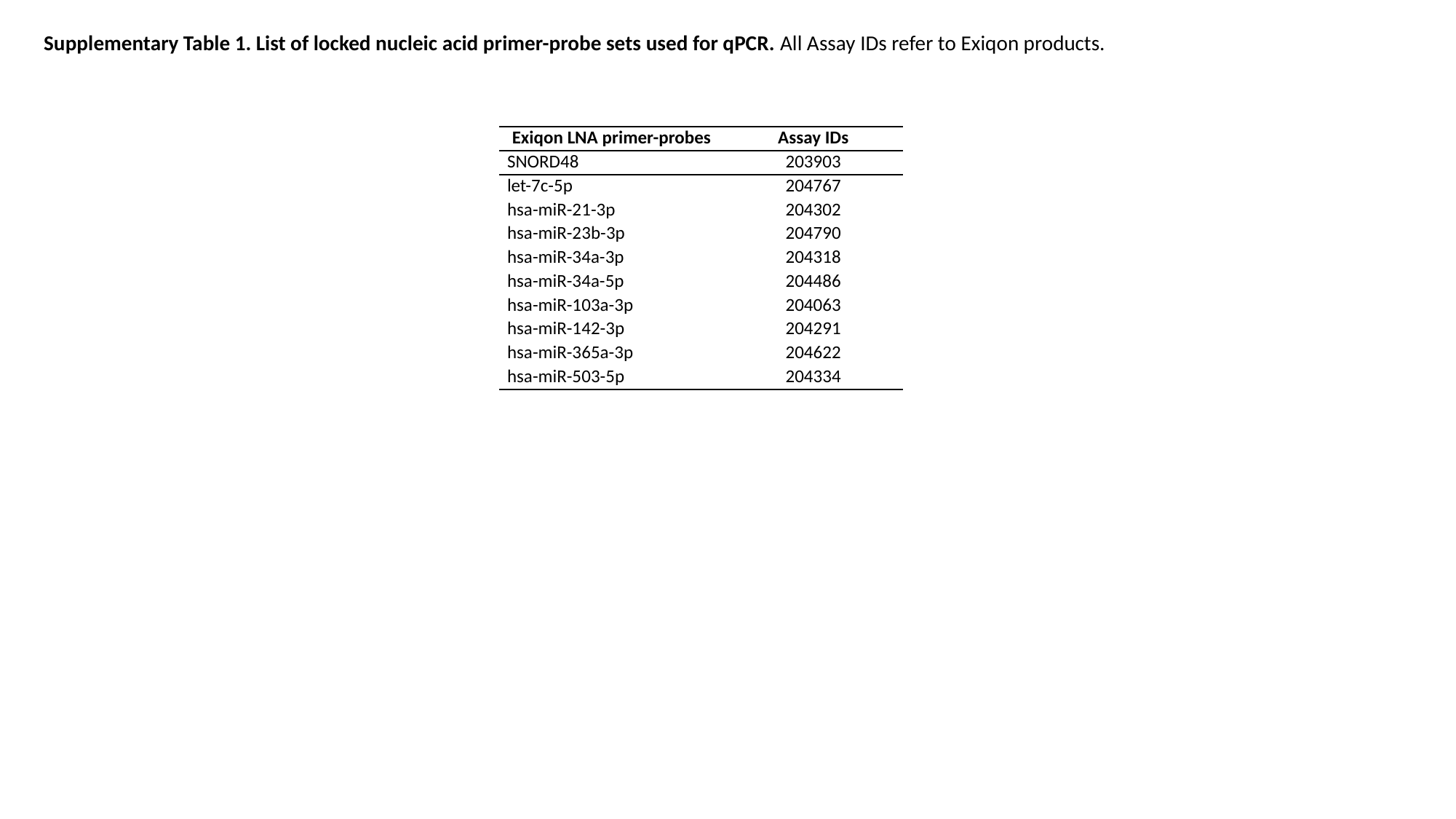

Supplementary Table 1. List of locked nucleic acid primer-probe sets used for qPCR. All Assay IDs refer to Exiqon products.
| Exiqon LNA primer-probes | Assay IDs |
| --- | --- |
| SNORD48 | 203903 |
| let-7c-5p | 204767 |
| hsa-miR-21-3p | 204302 |
| hsa-miR-23b-3p | 204790 |
| hsa-miR-34a-3p | 204318 |
| hsa-miR-34a-5p | 204486 |
| hsa-miR-103a-3p | 204063 |
| hsa-miR-142-3p | 204291 |
| hsa-miR-365a-3p | 204622 |
| hsa-miR-503-5p | 204334 |

## Slide 2
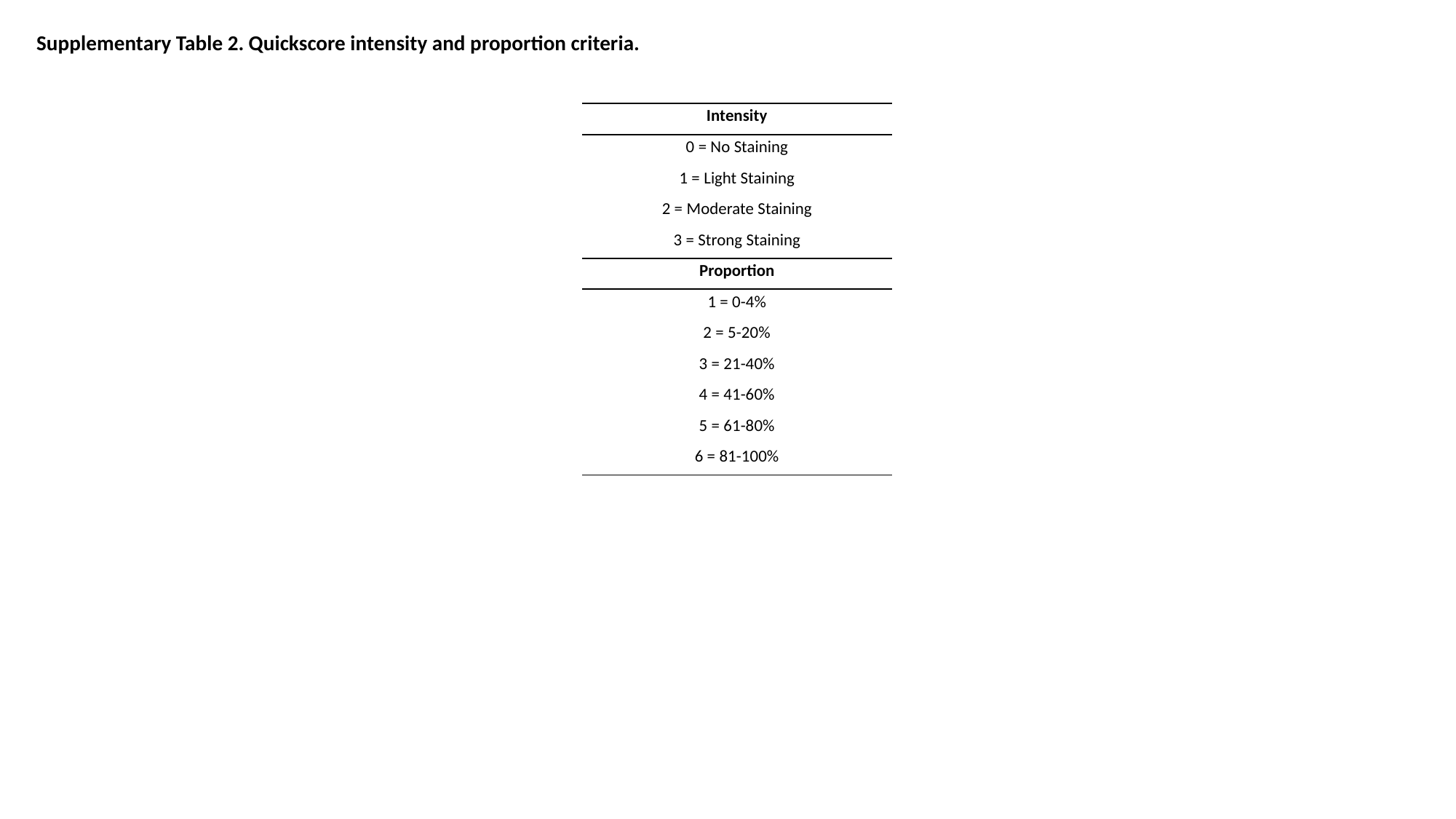

Supplementary Table 2. Quickscore intensity and proportion criteria.
| Intensity |
| --- |
| 0 = No Staining |
| 1 = Light Staining |
| 2 = Moderate Staining |
| 3 = Strong Staining |
| Proportion |
| 1 = 0-4% |
| 2 = 5-20% |
| 3 = 21-40% |
| 4 = 41-60% |
| 5 = 61-80% |
| 6 = 81-100% |

## Slide 3
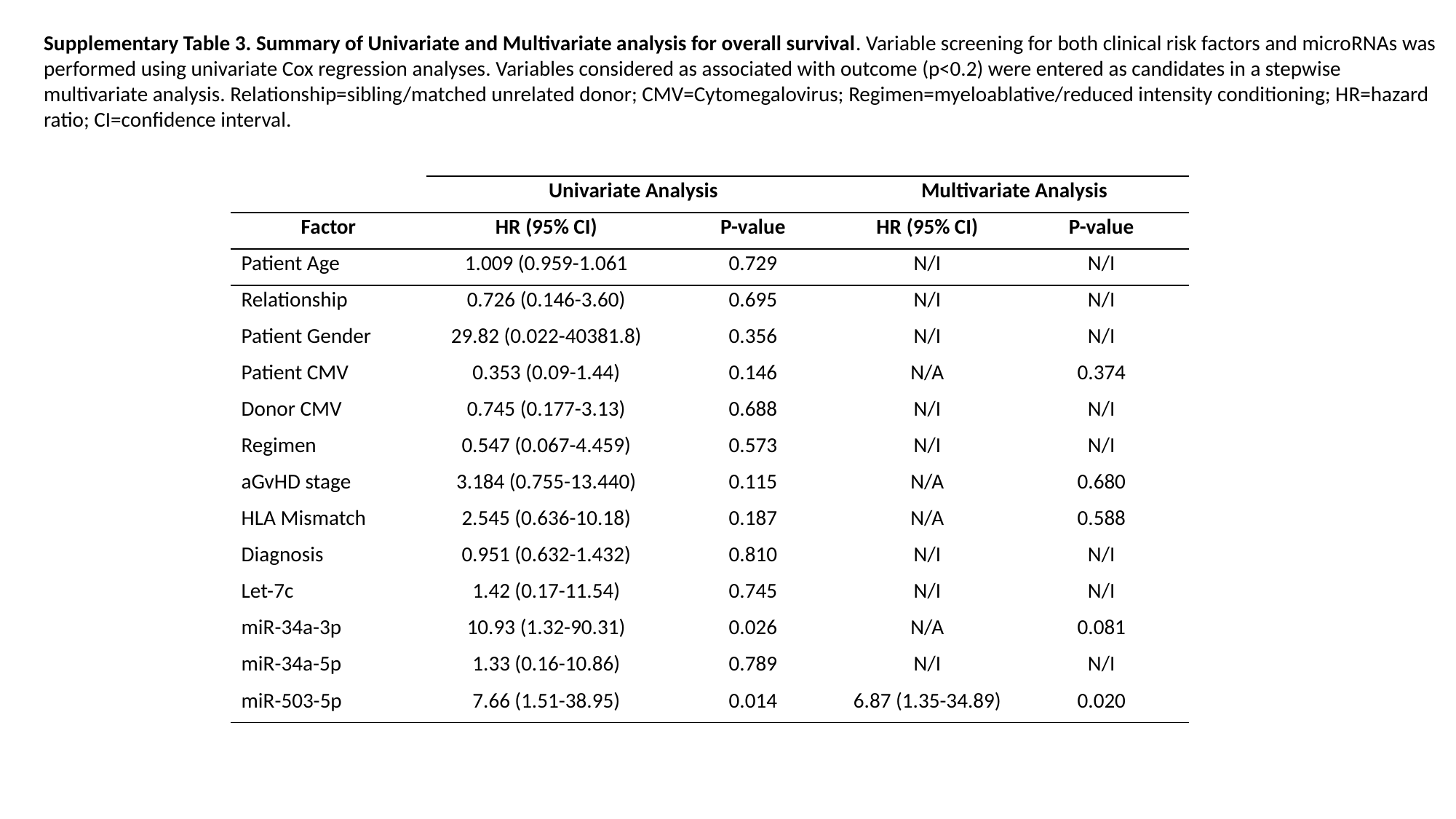

Supplementary Table 3. Summary of Univariate and Multivariate analysis for overall survival. Variable screening for both clinical risk factors and microRNAs was performed using univariate Cox regression analyses. Variables considered as associated with outcome (p<0.2) were entered as candidates in a stepwise multivariate analysis. Relationship=sibling/matched unrelated donor; CMV=Cytomegalovirus; Regimen=myeloablative/reduced intensity conditioning; HR=hazard ratio; CI=confidence interval.
| | Univariate Analysis | | Multivariate Analysis | |
| --- | --- | --- | --- | --- |
| Factor | HR (95% CI) | P-value | HR (95% CI) | P-value |
| Patient Age | 1.009 (0.959-1.061 | 0.729 | N/I | N/I |
| Relationship | 0.726 (0.146-3.60) | 0.695 | N/I | N/I |
| Patient Gender | 29.82 (0.022-40381.8) | 0.356 | N/I | N/I |
| Patient CMV | 0.353 (0.09-1.44) | 0.146 | N/A | 0.374 |
| Donor CMV | 0.745 (0.177-3.13) | 0.688 | N/I | N/I |
| Regimen | 0.547 (0.067-4.459) | 0.573 | N/I | N/I |
| aGvHD stage | 3.184 (0.755-13.440) | 0.115 | N/A | 0.680 |
| HLA Mismatch | 2.545 (0.636-10.18) | 0.187 | N/A | 0.588 |
| Diagnosis | 0.951 (0.632-1.432) | 0.810 | N/I | N/I |
| Let-7c | 1.42 (0.17-11.54) | 0.745 | N/I | N/I |
| miR-34a-3p | 10.93 (1.32-90.31) | 0.026 | N/A | 0.081 |
| miR-34a-5p | 1.33 (0.16-10.86) | 0.789 | N/I | N/I |
| miR-503-5p | 7.66 (1.51-38.95) | 0.014 | 6.87 (1.35-34.89) | 0.020 |

## Slide 4
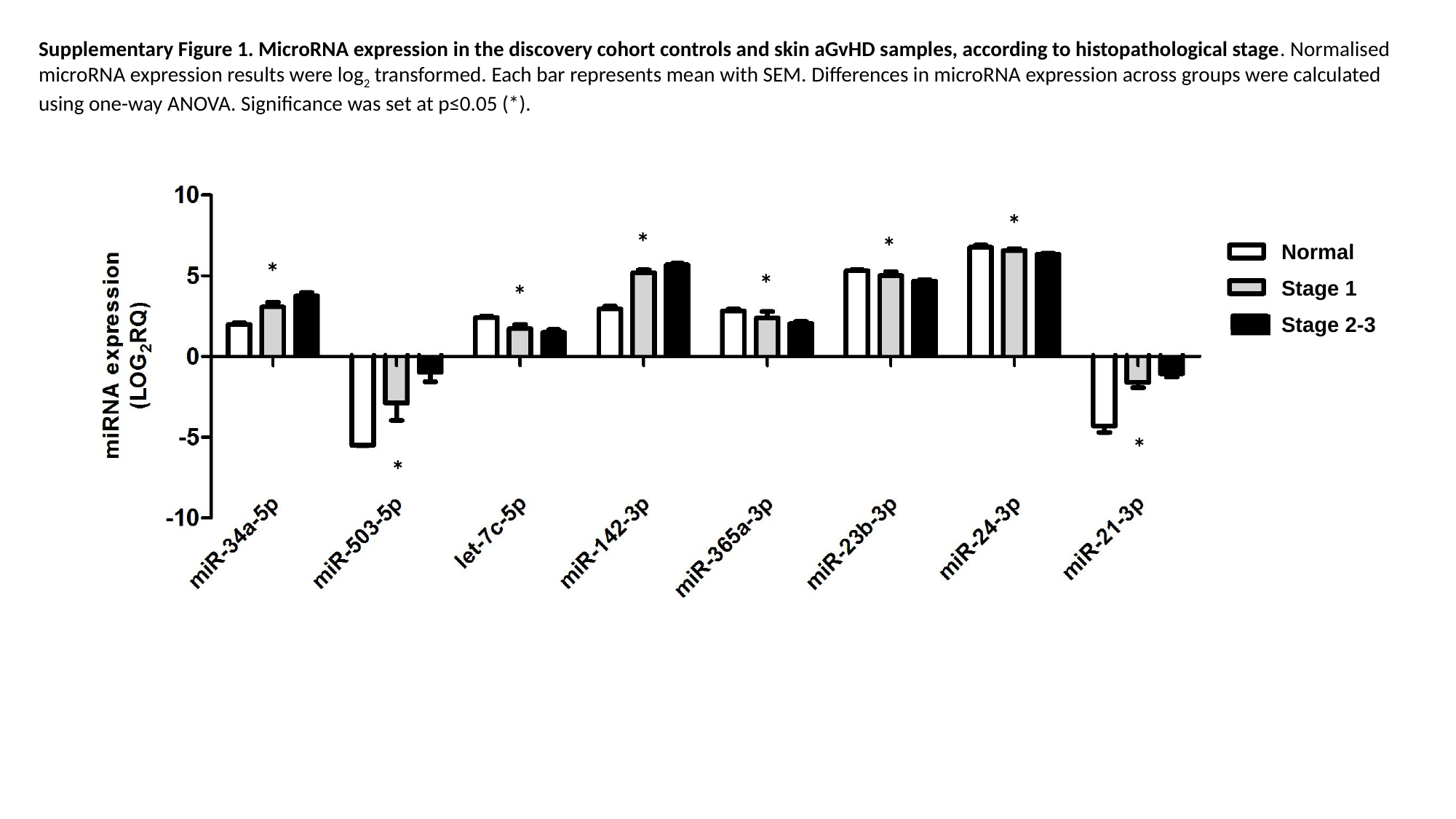

Supplementary Figure 1. MicroRNA expression in the discovery cohort controls and skin aGvHD samples, according to histopathological stage. Normalised microRNA expression results were log2 transformed. Each bar represents mean with SEM. Differences in microRNA expression across groups were calculated using one-way ANOVA. Significance was set at p≤0.05 (*).
*
*
*
*
*
*
*
*
Normal
Stage 1
Stage 2-3

## Slide 5
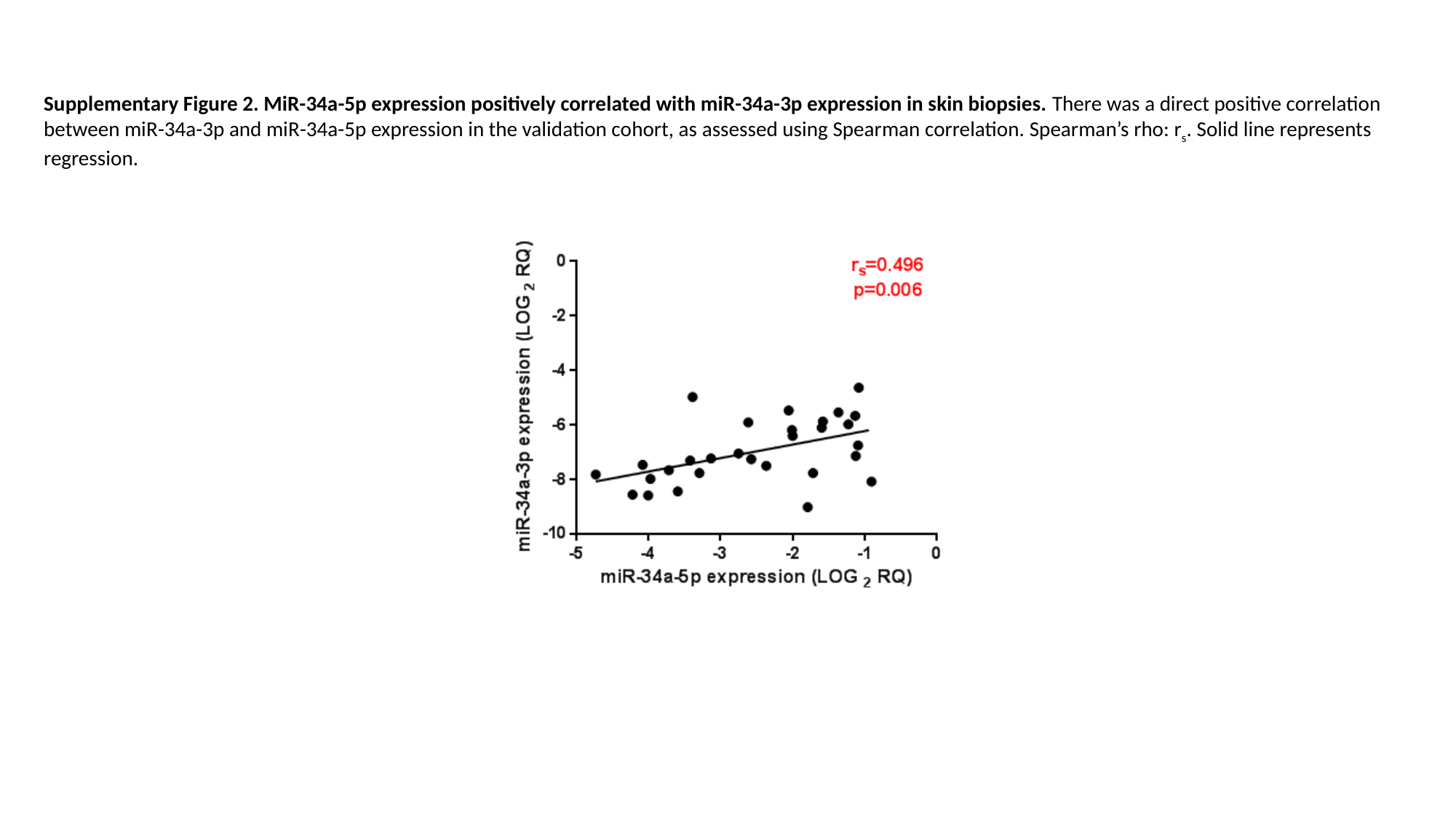

Supplementary Figure 2. MiR-34a-5p expression positively correlated with miR-34a-3p expression in skin biopsies. There was a direct positive correlation between miR-34a-3p and miR-34a-5p expression in the validation cohort, as assessed using Spearman correlation. Spearman’s rho: rs. Solid line represents regression.

## Slide 6
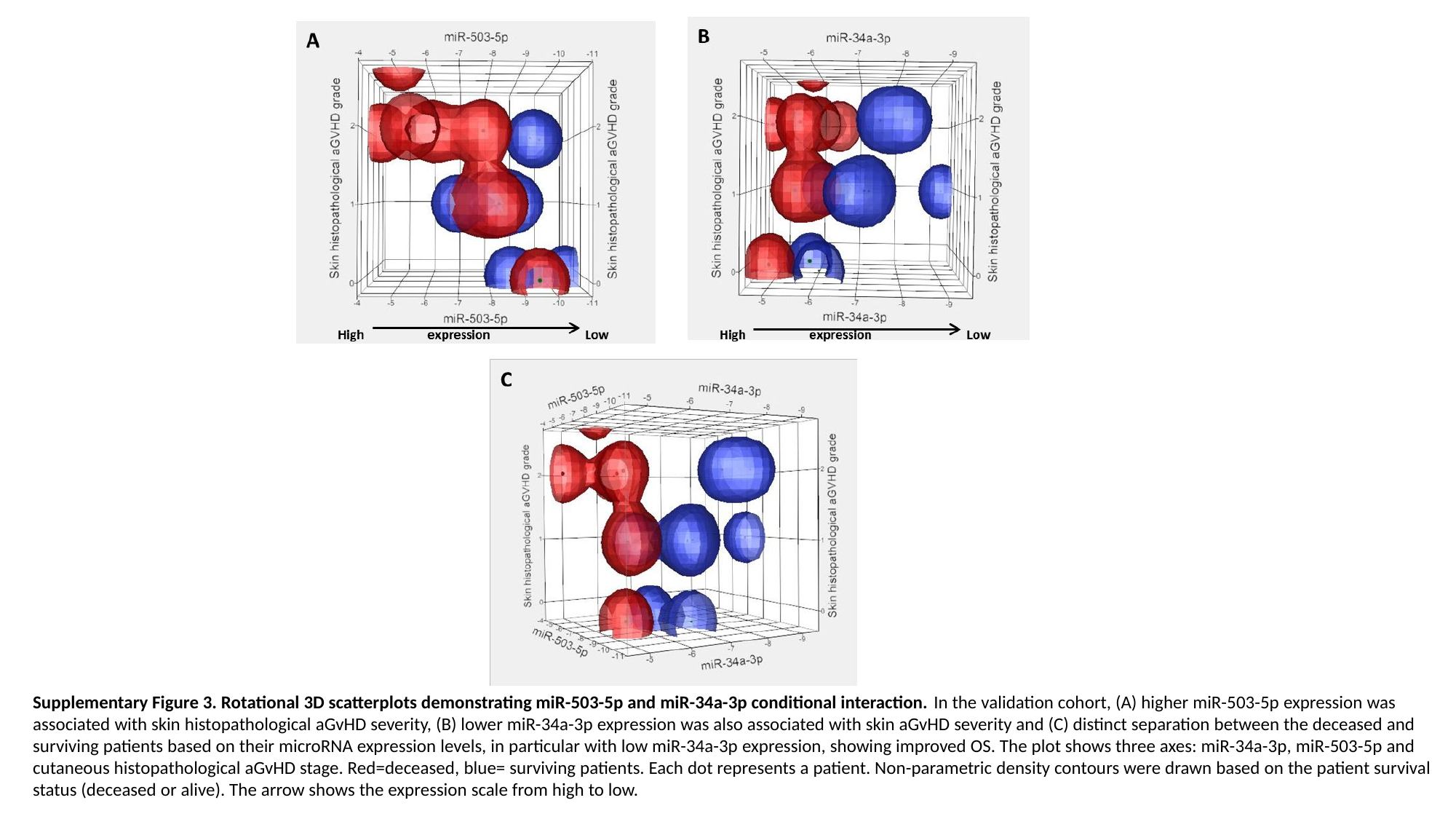

Supplementary Figure 3. Rotational 3D scatterplots demonstrating miR-503-5p and miR-34a-3p conditional interaction. In the validation cohort, (A) higher miR-503-5p expression was associated with skin histopathological aGvHD severity, (B) lower miR-34a-3p expression was also associated with skin aGvHD severity and (C) distinct separation between the deceased and surviving patients based on their microRNA expression levels, in particular with low miR-34a-3p expression, showing improved OS. The plot shows three axes: miR-34a-3p, miR-503-5p and cutaneous histopathological aGvHD stage. Red=deceased, blue= surviving patients. Each dot represents a patient. Non-parametric density contours were drawn based on the patient survival status (deceased or alive). The arrow shows the expression scale from high to low.

## Slide 7
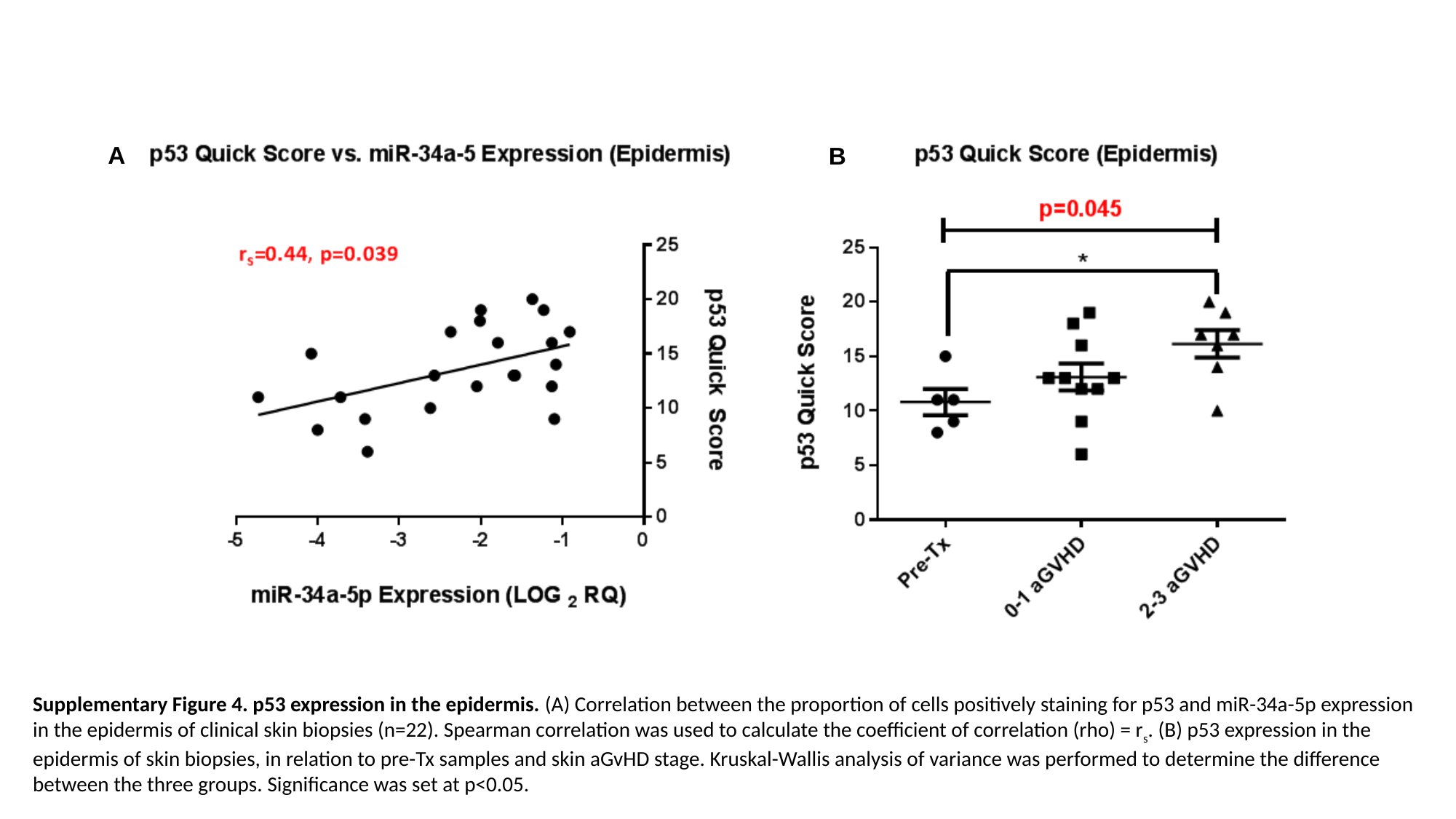

A
B
Supplementary Figure 4. p53 expression in the epidermis. (A) Correlation between the proportion of cells positively staining for p53 and miR-34a-5p expression in the epidermis of clinical skin biopsies (n=22). Spearman correlation was used to calculate the coefficient of correlation (rho) = rs. (B) p53 expression in the epidermis of skin biopsies, in relation to pre-Tx samples and skin aGvHD stage. Kruskal-Wallis analysis of variance was performed to determine the difference between the three groups. Significance was set at p<0.05.
